# Systematic characterization of novel lncRNAs responding to phosphate starvation in *Arabidopsis thaliana*

**DOI:** 10.1186/s12864-016-2929-2

**Published:** 2016-08-18

**Authors:** Jiapei Yuan, Ye Zhang, Jinsong Dong, Yuzhe Sun, Boon L. Lim, Dong Liu, Zhi John Lu

**Affiliations:** 1 MOE Key Laboratory of Bioinformatics, Center for Plant Biology, Center for Synthetic and Systems Biology and Tsinghua-Peking Joint Center for Life Sciences, School of Life Sciences, Tsinghua University, Beijing, 100084 China; 2 School of Biological Sciences, the University of Hong Kong, Pokfulam Road, Hong Kong, China

**Keywords:** Long ncRNAs, RNA-Seq, Phosphate starvation, *Arabidopsis thaliana*, Poly(A)+, Poly(A)–

## Abstract

**Background:**

Previously, several long non-coding RNAs (lncRNAs) were characterized as regulators in phosphate (Pi) starvation responses. However, systematic studies of novel lncRNAs involved in the Pi starvation signaling pathways have not been reported.

**Results:**

Here, we used a genome-wide sequencing and bioinformatics approach to identify both poly(A) + and poly(A)– lncRNAs that responded to Pi starvation in *Arabidopsis thaliana*. We sequenced shoot and root transcriptomes of the Arabidopsis seedlings grown under Pi-sufficient and Pi-deficient conditions, and predicted 1212 novel lncRNAs, of which 78 were poly(A)– lncRNAs. By employing strand-specific RNA libraries, we discovered many novel antisense lncRNAs for the first time. We further defined 309 lncRNAs that were differentially expressed between P+ and P– conditions in either shoots or roots. Through Gene Ontology enrichment of the associated protein-coding genes (co-expressed or close on the genome), we found that many lncRNAs were adjacent or co-expressed with the genes involved in several Pi starvation related processes, including cell wall organization and photosynthesis. In total, we identified 104 potential lncRNA targets of PHR1, a key regulator for transcriptional response to Pi starvation. Moreover, we identified 16 candidate lncRNAs as potential targets of miR399, another key regulator of plant Pi homeostasis.

**Conclusions:**

Altogether, our data provide a rich resource of candidate lncRNAs involved in the Pi starvation regulatory network.

**Electronic supplementary material:**

The online version of this article (doi:10.1186/s12864-016-2929-2) contains supplementary material, which is available to authorized users.

## Backgrounds

Plants possess an elaborate physiological system to respond to external stimuli and stress conditions [1], including phosphorus (P) deficiency. As one of the major mineral macronutrients, P is essential for plant growth and development. It is an important structural element for many macromolecules and participates in many cellular activities, including energy transfer, photosynthesis (PS), carbon assimilation, and activity regulation of many critical enzymes [2]. Despite its outstanding effect on plant growth and crop productivity, inorganic phosphate (Pi), the predominant absorbable form of P for plant roots, is often insoluble and not utilizable for plant acquisition in most soils [3–6]. To cope with Pi deprivation, enhance Pi availability and maintain Pi homeostasis, plants have evolved a variety of adaptive strategies [7]. These strategies include remobilization and redistribution of internal P and enhanced assimilation of Pi from the environment [8–11]. Although these responses have been well characterized in many plant species, the underlying molecular mechanisms that regulate these responses remain largely unknown.

With the development of many genetic resources, several important transcription factors (TFs) have been identified during recent decades, including members of the MYB (PHR1 and MYB62), WRKY (WRKY75 and WRKY6), ZAT (ZAT6) and bHLH (bHLH32 and OsPTF1) families [12–18]. Among these TFs, PHR1 and its most closely related genes, PHL1 (PHR1-like 1) and PHL2, are central integrators in transcriptional regulation of Pi starvation responses [19, 20]. Genome-wide characterization demonstrated that the promoter regions of many Pi starvation responsive genes contain the P1BS element, which can be recognized and bound by PHR1, PHL1, and PHL2 [19–21]. On the other hand, at the post-transcriptional level, miRNA399 has been identified as a key regulator of Pi homeostasis in post-transcriptional regulation [22]. The expression of miR399 is highly induced in both shoots and roots by a decrease in external Pi levels [23, 24]. MiR399 cleaves *PHO2* mRNA, which encodes an ubiquitin E2 conjugase (UBC24). PHO2 has been demonstrated to regulate Pi uptake in roots and Pi translocation from roots to shoots by mediating protein degradation of high-affinity Pi transporters and PHOSPHATE1 (PHO1) [25, 26]. Two Pi starvation-induced long non-coding RNAs, *IPS1* and *AT4*, further modulate the activity of miRNA399, through a mechanism called ‘target mimicry’ [27]. *IPS1*–miR399 matching would therefore lead to the inhibition of miR399-mediated cleavage of *PHO2* transcripts, thus influencing downstream Pi uptake and translocation [27]. Additionally, in rice, a *cis*-nature antisense transcript of *OsPHO1;2* (*cis*-NAT_PHO1;2_) was shown to act as a translational enhancer of *OsPHO1;2* [28]. Some other plant long non-coding RNA (lncRNA) candidates were also reported as potent regulators mediating gene expression and protein recruitment during stress responses [29–31]. For instance, two well-investigated lncRNAs, COOLAIR [32] and COLDAIR [33], were found to be involved in repression of *FLC*, a key suppressor of vernalization-controlled flowering in Arabidopsis. COOLAIR and COLDAIR are antisense and sense to the *FLC* transcript on the genome, respectively. The crucial functions of the above three lncRNAs demonstrate that antisense and intronic lncRNAs have a great effect on the regulation of cognate gene expression [34]. It is still unclear, however, whether the Arabidopsis genome contains other lncRNAs that participate in the adaptive response to Pi starvation.

In addition to individual studies of signaling components involved in Pi starvation responses, there have also been systematic studies using high-throughput array and sequencing data [21, 35–40]. For instance, it was suggested that roots and shoots are two independent regulons because of minor overlap between root and shoot transcriptomes [37]. Furthermore, a global characterization, based on a split-root system, classified the root transcripts response to local or systemic signals, respectively [36]. These transcriptomic analyses greatly improved our knowledge of protein-coding genes’ regulatory networks. In addition to protein-coding mRNAs, microRNAs can also function as key regulators of Pi starvation stress signaling [41]. A comprehensive expression profiling of Pi-responsive small RNAs advanced our understanding of the regulation of Pi homeostasis mediated by small RNAs [42]. Although coding genes and miRNAs have been systematically investigated in Pi starvation responses, there has been no genome-wide study to identify and characterize novel lncRNAs participating in the response pathways to Pi starvation of Arabidopsis.

To systematically identify and characterize novel lncRNAs responding to Pi starvation, we developed a sequencing and bioinformatics framework for *Arabidopsis thaliana.* We first sequenced the poly(A) enriched [poly(A)+] and poly(A) depleted [poly(A)–] RNA libraries in the root and shoot tissues of Arabidopsis seedlings, either grown under Pi-sufficient (P+) or Pi-deficient (P–) conditions. We then identified and characterized approximately 1200 novel lncRNAs using a bioinformatics pipeline. These novel lncRNAs, as well as known lncRNAs previously annotated in TAIR10, were grouped into six clusters according to their differential expression levels between root and shoot tissues. Furthermore, 104 and 16 lncRNAs were predicted as potential regulatory targets of PHR1 and miR399, respectively. Overall, our work provides an abundant resource of candidate lncRNAs associated with Pi starvation signaling pathways and enriched the regulatory network of Pi starvation responses in Arabidopsis.

## Results

### Genome-wide identification of novel lncRNAs in Arabidopsis under Pi deficiency

**Fig. 1:**
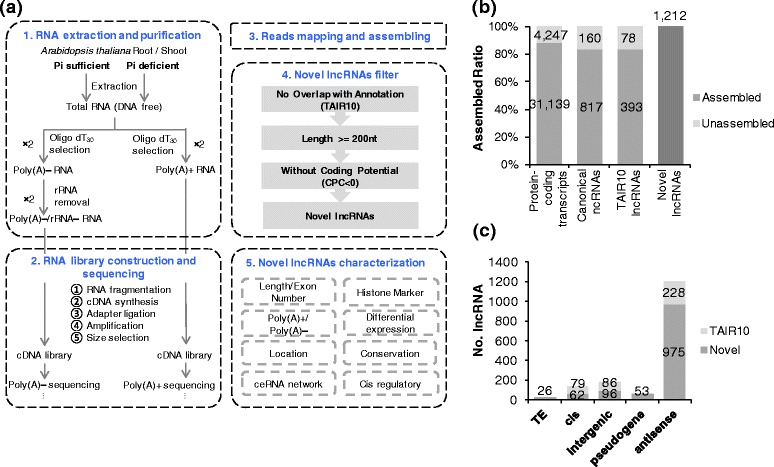
Flowchart of identification of lncRNAs responsive to Pi starvation in Arabidopsis. **a** 1. Plant treatment and poly(A) + and poly(A)– RNA extractions and purifications. 2. Construction of strand-specific cDNA libraries and sequencing. 3. RNA-Seq data mapping and assembly. 4. Novel lncRNAs were obtained after three filter steps, including overlap with annotation, calculation of length, and coding potential. 5. Characterization of novel lncRNAs at different levels, such as transcript length, exon number, polyadenylation, expression, epigenetic signature, and transcriptional and post-transcriptional regulation. **b** Assembled ratio of protein-coding transcripts and lncRNA transcripts. Approximately 90 % of protein-coding transcripts and over 80 % of TAIR10 lncRNAs could be completely assembled based on our RNA-Seq data. **c** Genomic positions of TAIR10 and novel lncRNAs. The majority of TAIR10 and novel lncRNAs were antisense to coding transcripts, and lncRNAs overlapped with TEs or pseudogenes accounted for a small fraction. Other lncRNAs with no overlap with any annotated coding transcripts or lncRNA were defined as intergenic or *cis*-lncRNAs according to the distance between lncRNAs and adjacent genes

To systematically identify lncRNAs that responded to Pi starvation, we performed strand-specific poly(A) + and poly(A)– RNA sequencing of 10-day-old Arabidopsis seedlings grown under P+ and P– conditions. We chose 10-day-old seedlings with obvious Pi starvation phenotype in order to include the long-term Pi starvation responsive lncRNAs (Additional file 1: Figure S1). Roots and shoots were separately collected from various plant samples with two biological replicates (see Methods). We obtained approximately 400 M pair-end reads, 94 % of which could be mapped to the Arabidopsis genome (TAIR10) (Additional file 2: Table S1). From these short reads, we assembled 22,972 new transcripts (Additional file 1: Figure S2a). Subsequently, 1212 novel lncRNA transcripts were identified using a bioinformatics pipeline (Fig. 1a, Additional file 3, see Methods). In addition to the novel transcripts, 90 % of the protein-coding genes and 83 % of the TAIR10 lncRNAs could be fully assembled with our RNA-Seq data (Fig. 1b). Besides, we compared our defined 1212 novel lncRNAs with lncRNAs collected by PlncDB (see Methods) [43]. We found that many of the antisense lncRNAs have overlapped with natural antisense transcripts (NATs), which were defined by previous studies based on EST and tilling array datasets [44–46] (Additional file 1: Figure S2b-d, Additional file 3).

We first compared the genomic positions of annotated (TAIR10) and novel lncRNAs (Fig. 1c). Because we used a strand-specific RNA library construction protocol, we were able to identify many (975) novel antisense lncRNAs. In addition, 79 and 62 of the TAIR10 and novel lncRNAs were defined as *cis*-lncRNAs, respectively, as they were close to (≤500 nt) protein-coding genes. We also found that 4 and 2 % of the novel lncRNAs overlapped with pseudogenes and transposable element (TE)-related genes, respectively. The remaining lncRNAs came from intergenic regions, and were not close or antisense to any protein-coding genes.

### Characterization of the TAIR10 and novel lncRNAs

**Fig. 2:**
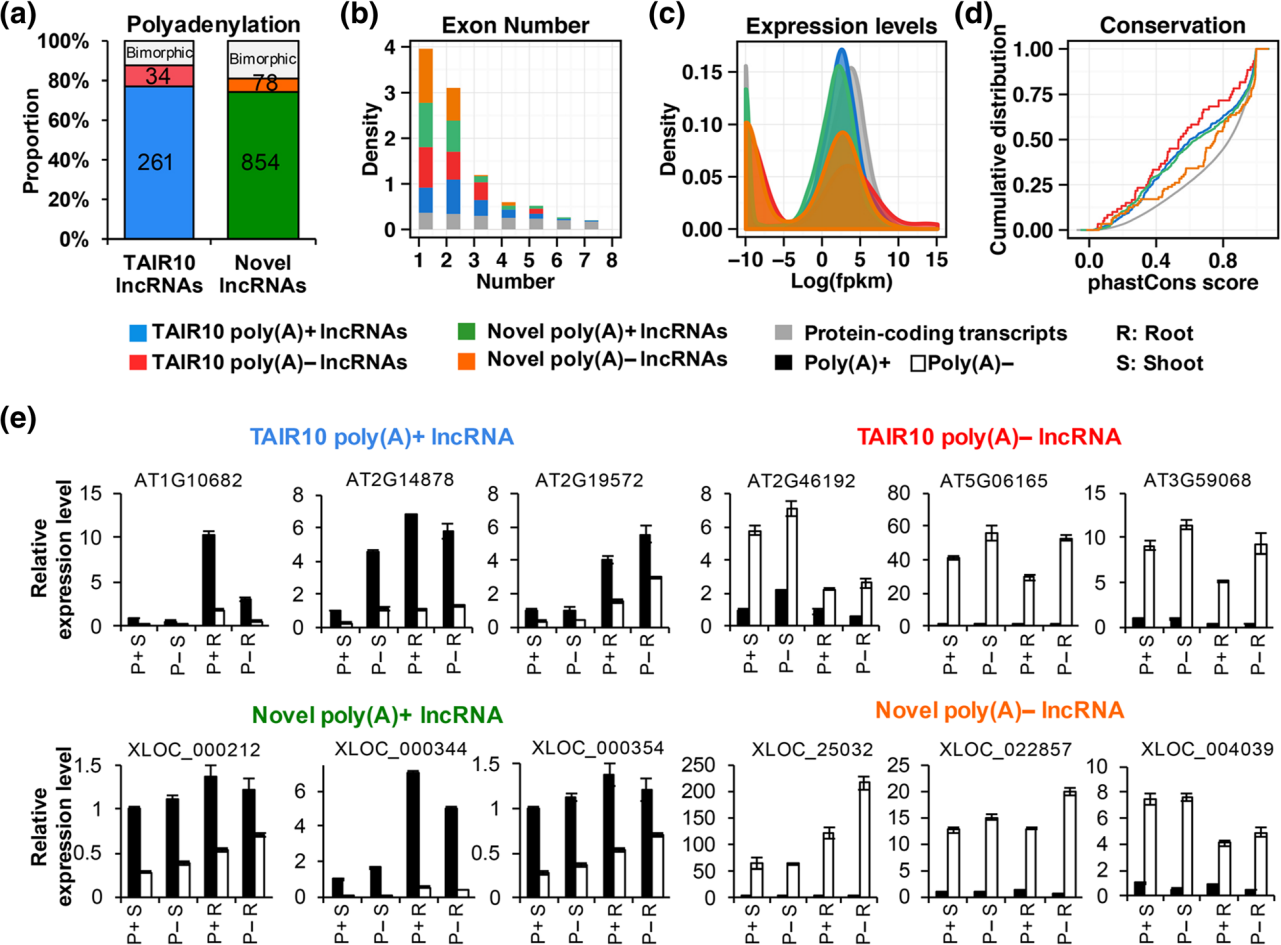
Polyadenylation and characterizations of lncRNAs. **a** Poly(A) + and poly(A)– proportions of TAIR10 and novel lncRNAs. There were 1115 lncRNAs (accounting for over 70 %) classified as poly(A) + lncRNAs, and 112 classified as poly(A)– lncRNAs. **b**–**d** Comparison of poly(A) + and poly(A)– lncRNAs using exon number, expression level, and conservation. The lncRNAs exhibited lower exon number and conservation than protein-coding transcripts. Poly(A)– lncRNAs showed lower expression levels than poly(A) + lncRNAs. **e** Validation of poly(A) + and poly(A)– lncRNAs using qRT-PCR. Poly(A) + lncRNAs were much more abundant in poly(A) + than poly(A)– samples; whereas, poly(A)– lncRNAs were mainly expressed in poly(A)– samples

We characterized various aspects of the TAIR10 and novel lncRNAs, including polyadenylation [Poly(A)] (Fig. 2a), exon number (Fig. 2b), expression level (Fig. 2c), and conservation (Fig. 2d), etc. (Additional file 1: Figures S3–S8).

We first classified all expressed transcripts into poly(A)+, poly(A)– and bimorphic groups according to their relative abundance in poly(A) + and poly(A)– samples from roots or shoots under the same condition (see Methods). The biomorphic transcripts were those not showing distinguishable expression differences between poly(A) + and poly(A)– RNA-Seq data. Over 70 % of the TAIR10 and novel lncRNAs were defined as poly(A) + transcripts (74 % in roots and 75 % in shoots) under P+ condition (Fig. 2a, Additional file 1: Figure S3). Moreover, lncRNAs had lower exon numbers and transcript lengths than protein-coding transcripts (Fig. 2b and Additional file 1: Figure S4, Additional file 2: Table S2). These observations were consistent with previous studies [29, 46], suggesting that alternative splicing events of protein-coding genes were more abundant than lncRNAs [47, 48]. In addition, protein-coding transcripts showed higher expression levels than lncRNAs; and poly(A) + transcripts were usually more abundant than poly(A)– transcripts (Fig. 2c and Additional file 1: Figure S5). Although the lncRNAs were usually expressed at low levels, their activity was still well supported by different histone markers and DNase signals (Additional file 1: Figure S6). Finally, we showed that lncRNAs were less conserved than protein-coding transcripts (Fig. 2d, Additional file 1: Figures S7 and S8), which were also in agreement with previous lncRNA studies [29, 49].

To test the accuracy of our poly(A) classification, the expression levels of candidate lncRNAs in poly(A) + and poly(A)– RNA samples were validated. We randomly selected eight candidates from each class of poly(A)+, poly(A)– and bimorphic lncRNAs for quantitative real-time PCR (qRT-PCR) validation. The qRT-PCR results confirmed all of the poly(A)+, seven poly(A)– and six biomorphic lncRNAs defined by RNA-Seq data; and 18 of the validated lncRNAs were presented (Fig. 2e and Additional file 1: Figure S9).

### The lncRNAs differentially expressed in Pi-deprived roots and shoots

**Fig. 3:**
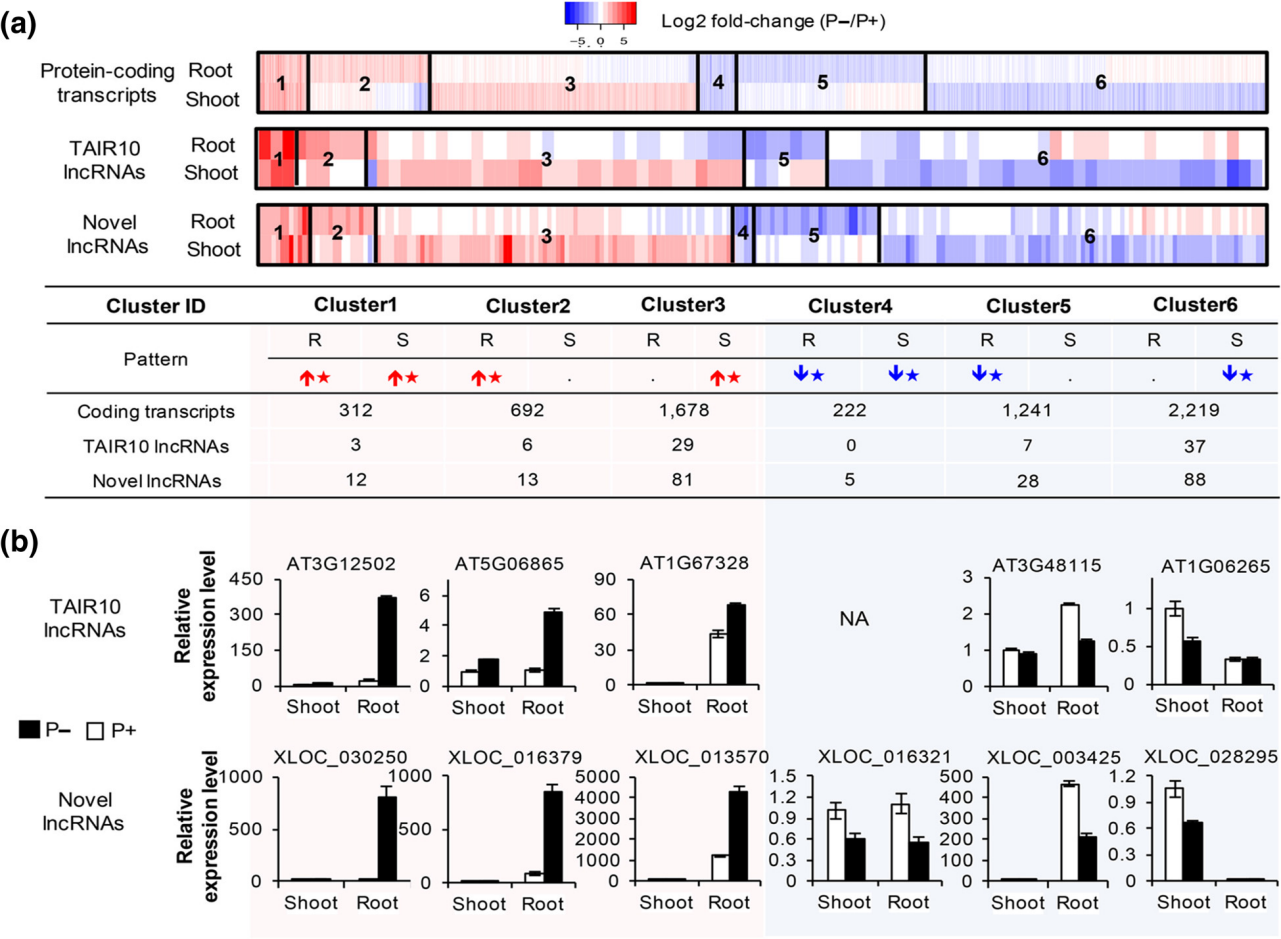
P– regulated lncRNAs revealed different response patterns in roots and shoots. **a** Heatmap and the number of differentially expressed protein-coding transcripts and lncRNAs of six clusters. In total, 6364 protein-coding transcripts, 82 TAIR10 lncRNAs, and 227 novel lncRNAs were identified as significantly differentially expressed under P– condition [fold change (P–/P+) > 2, *p-value* < 0.05 and fold change (P–/P+) < 0.5, *p-value* < 0.05]. Most of the P– responsive transcripts belonged to clusters 3 and 6, and were induced or repressed in shoots only. The red up arrows stand for up-regulated genes or lncRNAs and the blue down arrows represent the down-regulated ones. Asterisk indicates a significant difference [fold change (P–/P+) > 2, *p-value* < 0.05 and fold change (P–/P+) < 0.5, *p-value* < 0.05]. The *p* values were calculated with DESeq2. **b** Validation of lncRNAs from six clusters using qRT-PCR. TAIR10 and novel lncRNAs from six clusters were verified and their expression patterns were consistent with RNA-Seq

We calculated the differentially expressed transcripts under P+ and P– conditions, based on the RNA-Seq data (see Methods). In total, we identified 82 TAIR10 lncRNA and 227 novel lncRNA transcripts, which were significantly induced or repressed under Pi starvation condition (Additional file 4). We found some differentially expressed protein-coding transcripts that were consistent with previous studies (Additional file 1: Figure S10) [21, 35, 37, 50, 51]. Subsequently, we grouped these transcripts into six clusters according to the up-/down- regulation levels in roots and shoots (Fig. 3a): transcripts were induced or repressed significantly in both roots and shoots (clusters 1 and 4), roots only (clusters 2 and 5), and shoots only (clusters 3 and 6). Interestingly, clusters 3 and 6 contained most of the differentially expressed transcripts for both lncRNAs and protein-coding transcripts. This suggested that more RNAs were regulated in shoots than in roots by Pi starvation. Then, we randomly selected 16 TAIR10 and 19 novel lncRNAs from the above clusters for the differential expression validation using qRT-PCR. Except for the absence of TAIR10 lncRNAs in cluster 4, 10 TAIR10, and 12 novel candidate lncRNAs from other clusters were verified in roots and shoots (Fig. 3b and Additional file 1: Figure S11). Moreover, the lncRNAs with and without poly(A) tails were also counted for each cluster (Additional file 1: Figure S12), based on the previous polyadenylation classification (Fig. 2a).

We also calculated alternative splicing (AS) events for these lncRNAs, based on the RNA-Seq data (Additional file 1: Figure S13). The main patterns of AS were retained intron (RI) and alternative 3ʹ splice site (A3SS). Remarkably, the AS events were significantly enriched in lncRNAs from cluster 6 (χ^2^ test, *P* < 0.001). This result suggested that lncRNAs repressed in shoots (cluster 6) probably underwent regulation at both transcriptional and post-transcriptional levels, resulting in differential expression and AS under Pi starvation condition.

### Function and pathway prediction of lncRNAs that responded to Pi starvation

**Fig. 4:**
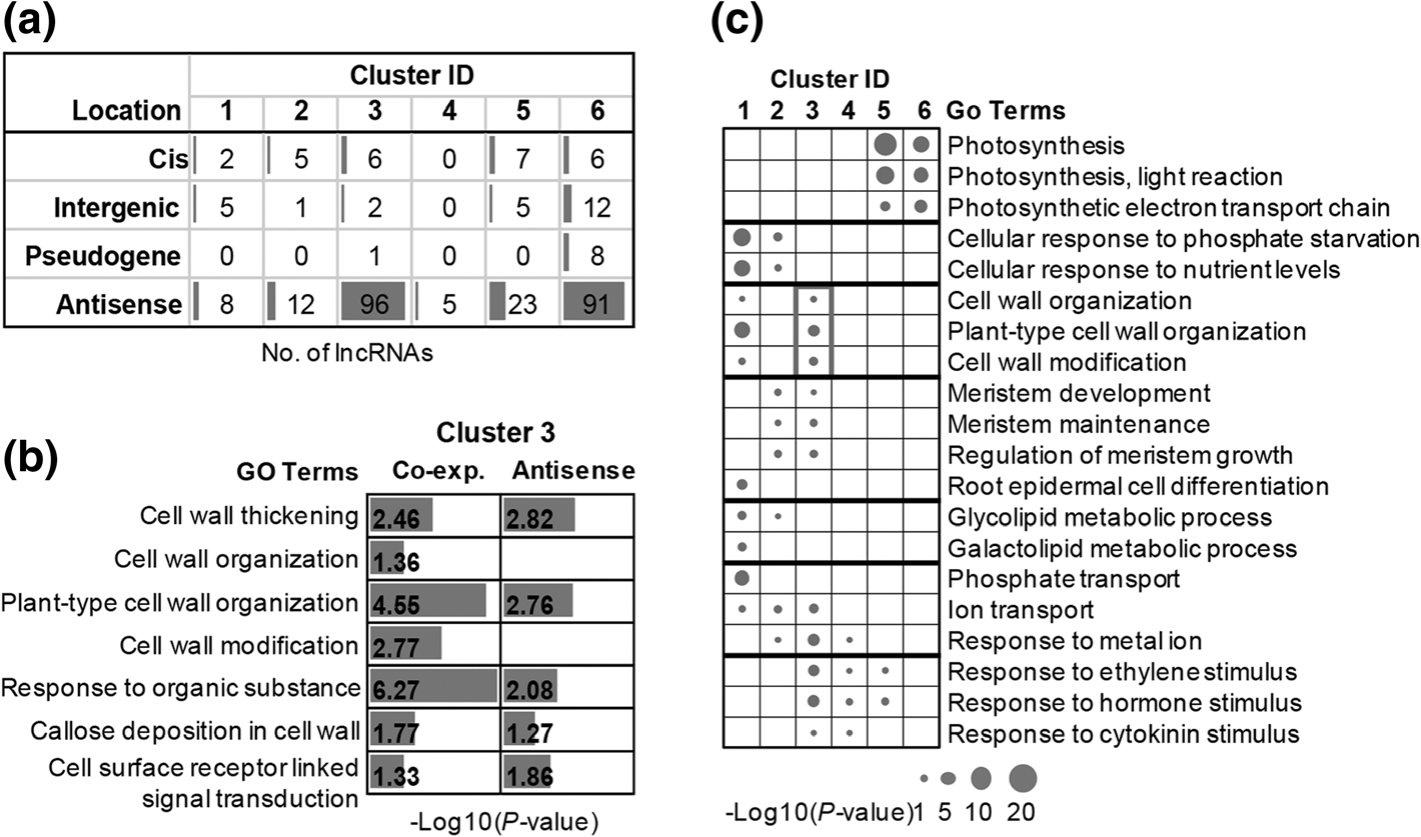
Functional prediction of lncRNAs by co-position and co-expression. **a** Genomic positions of lncRNAs (including both TAIR10 and novel lncRNAs) from six clusters. Differentially expressed lncRNAs were mainly antisense lncRNAs. **b** Functional predictions of lncRNAs of cluster 3 via co-localization and co-expression. Protein-coding genes that were antisense to lncRNAs of cluster 3 were enriched in GO terms related to cell wall organization, which was consistent with the enriched functions of protein-coding genes from cluster 3. **c** GO of protein-coding genes from six clusters. Protein-coding genes were enriched in many Pi starvation responsive biological processes, such as photosynthesis, morphology change, root cell differentiation, phosphate transport, and hormone response

We used two methods to associate and predict the potential functions of lncRNAs differentially expressed under Pi starvation: genomic position and expression pattern defined by the above six clusters. First, we tried to predict the functions of lncRNAs by linking them to their adjacent protein-coding genes on the chromosome (Fig. 4a). The antisense lncRNAs and *cis-*lncRNAs (close and on the sense strand) could serve as *cis*-regulatory elements to regulate the related protein-coding genes [32]. Consistent with the previous pattern (Fig. 1c), most of the differentially expressed lncRNAs in the above six clusters were antisense lncRNAs; and the antisense lncRNAs were mainly distributed in clusters 3 and 6 (Fig. 4a). Based on the Gene Ontology (GO) enrichment analyses, we found that protein-coding genes antisense to the up-regulated lncRNAs in cluster 3 (shoots only) were mainly related to cell wall thickening and cell surface signal transduction (Fig. 4b), which were important processes of Pi starvation responses [35].

In addition to utilizing the *cis*-regulatory relationships between lncRNAs and protein-coding genes, we then used the expression pattern to search the enriched functions. We found that the differentially expressed protein-coding genes in cluster 3 were also enriched with GO terms related to cell wall organization and modification (Fig. 4b). In other words, we found similar protein-coding genes using either genomic position relationship or co-expression pattern, suggesting that some differentially expressed lncRNAs and protein-coding genes in cluster 3 were antisense to each other. Some other Pi starvation-responsive GO terms were also revealed in other clusters (Fig. 4c). For instance, the up-regulated genes (clusters 1–3) had crucial roles in morphological processes, glycolipid metabolism, and ion transport. Additionally, some GO terms responding to nutrient starvation were enriched in clusters 1 and 2. Moreover, we found that 149 of 152 genes were co-expressed with their antisense lncRNAs significantly between P+ and P– conditions (Additional file 5).

More interestingly, many down-regulated genes in clusters 5 (roots only) and 6 (shoots only) were enriched in the PS-related GO terms (Fig. 4c). Thus, representative genes for PS from cluster 5 and 6, and their antisense lncRNAs (Additional file 6) from the same cluster were highlighted on the PS map (Additional file 1: Figure S14). That is, the labeled lncRNAs and coding genes in the map not only shared the same expression pattern (clusters 5 and 6), but were also antisense to each other. In general, representative genes for PS were greatly down-regulated by Pi deficiency in both roots and shoots. Additionally, the expression levels of many PS-related genes were more heavily suppressed in roots than in shoots (Additional file 1: Figure S15), and that is supported by previous studies [21, 35, 39, 50]. Comparing the suppressed PS genes in roots (cluster 5) and shoots (cluster 6) showed a universal decline in photosystem II. In contrast, the genes involved in photosystem I were more suppressed in roots than shoots.

**Fig. 5:**
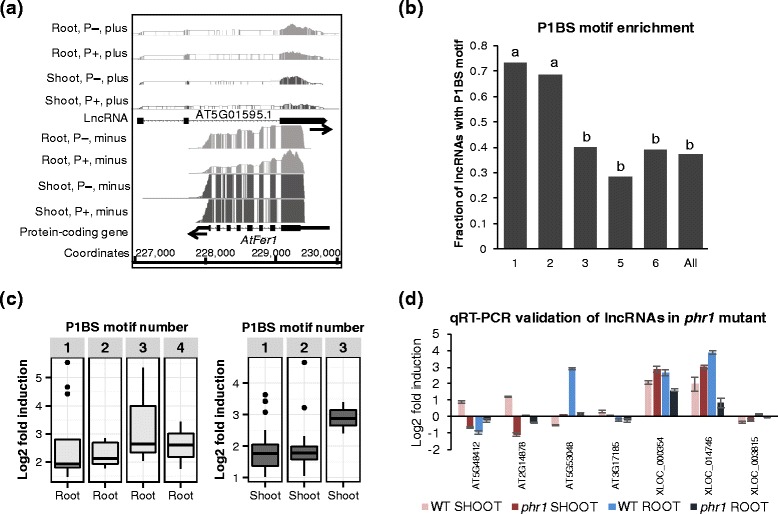
P– regulated lncRNAs were potential targets of PHR1. **a** Integrated Genome Browser (IGB) visualization of expression level of lncRNA AT5G01595.1 and its antisense gene *AtFer1*. Both AT5G01595.1 and *AtFer1* were induced in roots under P– condition and carried a P1BS motif at their promoter regions. **b** P1BS motifs were significantly enriched for lncRNAs in clusters 1 and 2. The difference in ratios was tested using χ^2^: **a** compared with **b**, *P* < 0.01; no significant difference within **a** and **b. All**: All lncRNAs (1692) as control. **c** Correlation of P1BS motif number and fold induction of lncRNAs in roots and shoots. With increased P1BS content, fold induction of lncRNAs induced by Pi starvation also increased. The header numbers in the gray box indicate the P1BS motif number at the promoter regions of lncRNAs. **d** Validation of PHR1 potential targeted lncRNAs in the *phr1* mutant by qRT-PCR. The lncRNAs that PHR1 targeted were less induced/repressed in *phr1* mutant

Another lncRNA-gene regulation example was a Pi starvation-induced (PSI) lncRNA in cluster 2 (roots only), AT5G01595.1, which was antisense to a protein-coding gene, *AtFer1. AtFer1* was reported to be a PSI gene that could be up-regulated by the well-known transcription factor, PHR1 [52]. Due to the strand specificity of our RNA-Seq data, we clearly detected the differential expression of these two transcripts (Fig. 5a). The mapped RNA-Seq reads on each strand showed that both AT5G01595.1 and *AtFer1* were induced by Pi deficiency. Furthermore, P1BS motifs (GNATATNC), which were recognized by PHR1 [12, 19], were identified at the promoter regions of both *AtFer1* and AT5G01595.1. Moreover, qRT-PCR verified that *AtFer1* and AT5G01595.1 were less induced in the *phr1* mutant (Additional file 1: Figure S16). All of the above indicated that AT5G01595.1 was probably directly regulated by PHR1, and was involved in the Pi starvation signaling pathway.

### Pi starvation-responsive lncRNAs were preferentially regulated by PHR1 in roots

In addition to the above example of AT5G01595.1, we examined whether all the differentially expressed lncRNAs contained P1BS motifs at their promoter regions (Table 1 and Additional file 7). We defined the upstream 2-kb of transcripts as their promoters, and used FIMO [53] to find the P1BS sequence motifs (see Methods). The P1BS motif was significantly enriched in the promoters of the up-regulated lncRNAs (Fig. 5b) and protein-coding transcripts (Additional file 1: Figure S17) from clusters 1 (both roots and shoots) and 2 (roots only) (χ^2^ test, *P* < 0.01). Particularly, 73 and 68 % of the lncRNAs from clusters 1 and 2 contained P1BS motifs in their promoter regions, respectively. However, only 40 % of lncRNAs from cluster 3 (shoots only) contained P1BS motifs at promoters, and the fractions of lncRNAs containing a P1BS motif of clusters 5 and 6 (expressed in roots and shoots, respectively) were below 40 %. We chose all protein-coding transcripts (35,386 in total) and lncRNAs (1692 in total) as the background (controls) to survey the P1BS motif enrichment fraction at their promoter regions. We could see that motif prediction would generate many potential false positives, because the fractions of controls for both protein coding genes (Additional file 1: Figure S17a) and lncRNAs (Fig. 5b) tended to be high (30–40 %). Therefore, we added other supporting evidences (i.e., DNase data and transcriptional response to Pi starvation) as other filters to predict the PHR1’s targets (Additional file 7) (see Methods), which could remove about 80 % of the positives predicted by motif only.

**Table 1:** P1BS motif of PHR1 targeted protein-coding genes and lncRNAs

GeneID/Name	No. Motif	Position of P1BS motif^b^	Motif sequence	Cluster ID^c^
*AtPAP10* ^a^	1	-122	GAATATTC	2
*PAP1* ^a^	2	-656,-191	GCATATAC,GAATATCC	1
*AtACP5* ^a^	1	-288	GAATATCC	1
*AtSPX1* ^a^	3	-124,-881,-100	GGATATTC,GAATATTC,GAATATTC	1
*SQD1* ^a^	1	-663	GAATATGC	1
*AtIPS1* ^a^	2	-763,-729	GCATATTC,GCATATTC	1
*SQD2* ^a^	3	-188,-136,-572	GCATATGC,GTATATCC,GAATATTC	2
*At4* ^a^	2	-1028,-490	GTATATGC,GCATATTC	1
*RNS1* ^a^	1	-188	GTATATAC	1
*AtPT1* ^a^	3	-1301,-1283,-200	GGATATTC,GCATATTC,GTATATAC	3
*AtPT2* ^a^	3	-394,-388,-1349	GAATATGC,GCATATAC,GAATATAC	1
AT5G53048	1	-142	GTATATTC	1
AT1G21529	1	-81	GAATATCC	1
AT3G57157	2	-1760,-240	GCATATGC,GAATATCC	2
AT5G06865	2	-1673,-1199	GAATATTC,GTATATTC	2
AT5G01595	1	-1988	GCATATTC	2
XLOC_026999	3	-372,-139,-128	GCATATTC,GCATATTC,GAATATTC	1
XLOC_001691	3	-1391	GTATATAC	1
XLOC_013732	3	-39,-299,-150	GCATATTC,GAATATAC,GAATATTC	1
XLOC_030250	1	-69	GAATATTC	1
XLOC_025622	2	-243,-273	GTATATCC,GAATATAC	1
XLOC_004968	1	-183	GAATATGC	1
XLOC_032181	4	-427,-108,-1483,-79	GGATATCC,GGATATGC,GAATATCC,GGATATTC	2
XLOC_006489	2	-1453,-191	GCATATGC,GCATATTC	2
XLOC_010338	2	-1706,-488	GCATATCC,GTATATCC	2
XLOC_013570	1	-1796	GAATATTC	2
XLOC_005614	2	-588,-113	GCATATCC,GAATATTC	2
XLOC_013661	1	-204	GTATATTC	2
XLOC_014746	1	-62	GAATATGC	2
XLOC_015182	1	-78	GAATATCC	2

Note:

^a^Gene names listed in italics are Pi starvation induced protein-coding marker genes

^b^For protein-coding genes, the position is given for the most 5′-upstream nucleotide relative to the first ATG in the transcribed region. For lncRNAs, the position refers to the most 5′-upstream nucleotide relative to first nucleotide of transcripts

^c^This table only lists targeted lncRNAs in cluster 1 and 2. Full table of PHR1 targeted genes and lncRNAs are listed in Additional file 7

Moreover, we tested whether the numbers of P1BS sequence motifs at the promoter regions were correlated with differential expression levels of PHR1-targeted transcripts. We correlated the average number of P1BS motifs per transcript with the targets’ expression fold-change induced by Pi starvation, for both up-regulated lncRNAs (Fig. 5c) and protein-coding transcripts (Additional file 1: Figure S18). We found that when P1BS motif number increased so too did the induction of lncRNAs in roots and shoots. Furthermore, we also observed that the positions of P1BS motifs tended to be closer to their targeted genes/lncRNAs from cluster 1 than target transcripts from other clusters (Additional file 1: Figure S19).

Finally, we randomly selected three protein-coding genes and five candidate lncRNAs with P1BS motifs at their promoter regions, and validated their expression change in the *phr1* mutant using qRT-PCR. Both lncRNAs (Fig. 5d) and protein-coding genes (Additional file 1: Figures S20 and S21) showed lower fold-changes in the *phr1* mutant than the control (Col-0) plant. We used two lncRNAs (AT3G17185 and XLOC_003815) with P1BS motifs but no expression changes as negative controls, and found their expression levels were not affected by PHR1 (Fig. 5d). These results demonstrated that the candidate lncRNAs associated with P1BS motifs were very likely regulated by PHR1 under Pi starvation condition.

### Pi starvation-responsive lncRNAs targeted by miR399

MiR399 has previously been shown to be a crucial post-transcriptional regulator [54], and has been demonstrated to bind the mRNAs of the *PHO2* and *AT4/IPS1* lncRNA family [27, 55]. However, its targets at genomic scale are unknown, and long noncoding RNAs have been proven to serve as potential target mimics for miRNAs in plants [56]. Thus, we first used two small public RNA-Seq data sets [42, 57] to profile differentially expressed miRNAs under P+ and P– conditions (Additional file 1: Figure S22). In total, 13 and 11 miRNAs were significantly up-regulated in roots and shoots, respectively.

**Fig. 6:**
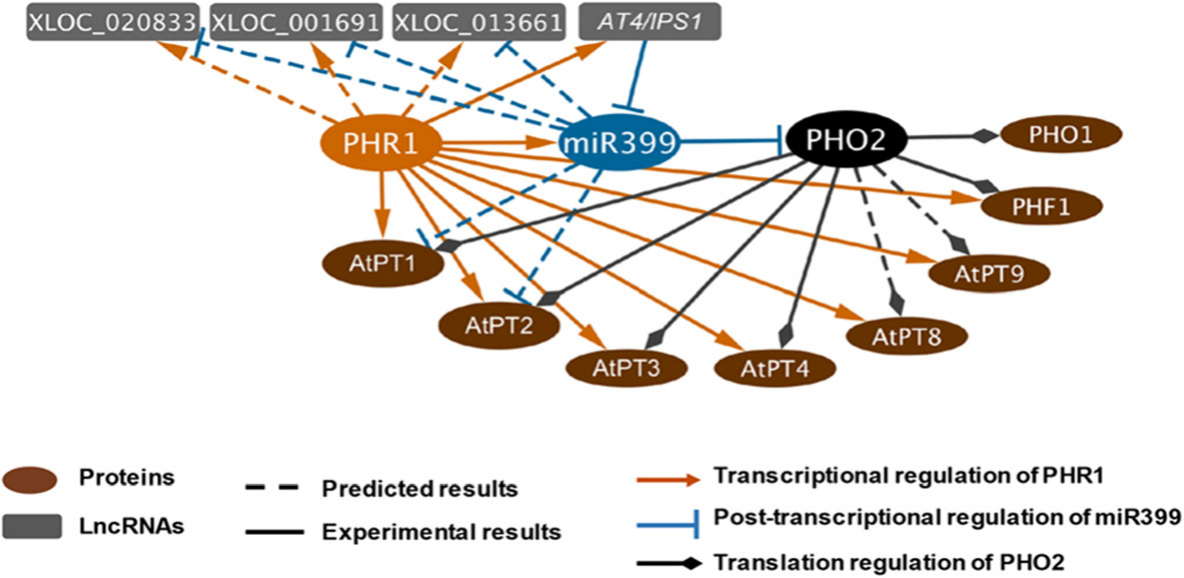
Extended regulatory network of PHR1, miR399, PHO2, and their targets. We extended the regulatory network of PHR1 and miR399 by P1BS motif enrichment prediction and ceRNA network construction. Many other protein-coding genes and lncRNAs showed potential to be regulated by PHR1 and miR399

Next, we predicted the potential targets of these differentially expressed miRNAs using psRobot [58]. We combined expression correlations of miR399 and its potential targets to obtain a competing endogenous RNA (ceRNA) network for miR399. The lncRNAs whose expression levels were negatively correlated with miR399 were shown in Table 2. Three lncRNAs (XLOC_020833, XLOC_001691 and XLOC_013661) were revealed to be potential targets of both PHR1 and miR399, indicating their feasible functions involved in the Pi starvation regulatory network (Tables 1 and 2, Fig. 6, and Additional file 7). There were a total of 42 potential targets of miR399 (Table 2), of which 16 were lncRNAs.

**Table 2:** Correlation between miR399 and potential target genes

miRNA	Target	Function description of target^a^	PCC^b^	Target score^c^	Cluster ID^d^
miR399a	*AtPT1*	Inorganic phosphate transporter 1–1	-0.72	5	3
miR399a	*SMXL5*	Clp amino terminal domain-containing protein	-0.72	5	1
miR399a	*ECT2*	Physically interacts with CIPK1.	-0.71	4.5	3
miR399a	*AtCS-C*	Dehydratase epimerase/racemase deaminase	-0.70	4.8	2
miR399a	*TIP4;1*	Aquaporin TIP4-1, transporter	-0.68	4.8	3
miR399a	*AtPT2*	Inorganic phosphate transporter 1-2	-0.67	5	1
miR399a	*SPPL5*	Signal peptide peptidase-like 5, aspartic protease	-0.67	5	6
miR399a	*AtSCAR4*	Protein SCAR4	-0.62	5	6
miR399a	*AtPP2-A5*	Protein PHLOEM PROTEIN 2-LIKE A5	-0.59	4	6
miR399a	AT3G18620	DHHC-type zinc finger family protein	-0.59	4.8	3
miR399a	*AtCFM2*	Involved in group I and group II intron splicing.	-0.59	4.5	6
miR399a	AT1G61860	Protein kinase family protein	-0.58	4.8	3
miR399a	AT2G31150	ATP binding / ATPase	-0.58	5	6
miR399a	AT3G44820	Phototropic-responsive NPH3 family protein	-0.58	4.8	3
miR399a	*IPGAM1*	2,3-bisphosphoglycerate-independent phosphoglycerate mutase 1	-0.57	5	3
miR399a	AT2G25420	mRNA splicing factor esterase kinase inhibitor	-0.57	4	6
miR399a	*AtOPR1*	12-oxophytodienoate reductase 1	-0.57	4.8	3
miR399a	AT4G19520	TIR-NBS-LRR class disease resistance protein	-0.56	5	6
miR399a	*OZS2*	Pectinesterase/pectinesterase inhibitor 3	-0.55	4.8	3
miR399a	AT2G38740	Phosphatase	-0.55	4.5	2
miR399a	*BLH1*	BEL1-like homeodomain protein 1	-0.53	4.2	3
miR399a	*TGA7*	Transcription factor TGA7	-0.51	5	3
miR399a	AT1G61590	Protein kinase superfamily protein	-0.51	4.8	3
miR399a	*CYP705A30*	Cytochrome P450, family 705, subfamily A, polypeptide 30	-0.44	2	3
miR399a	*PHO2*	Mediates degradation of PHO1 and PHT1s	-0.37	0.8	/
miR399a	*NF-YA10*	Nuclear transcription factor Y subunit A-10	-0.10	2.5	1
miR399d	XLOC_001691	/	-0.74	5	1
miR399b	XLOC_026270	/	-0.68	5	6
miR399e	XLOC_013661	/	-0.64	5	2
miR399b	XLOC_013840	/	-0.63	4.5	6
miR399e	XLOC_026270	/	-0.62	4.5	6
miR399e	XLOC_003248	/	-0.60	5	3
miR399e	XLOC_001806	/	-0.59	4.8	5
miR399a	XLOC_018338	/	-0.58	4.5	5
miR399d	XLOC_027498	/	-0.58	4.8	6
miR399d	XLOC_010238	/	-0.57	4.8	3
miR399c-5p	XLOC_019368	/	-0.57	5	4
miR399c-3p	XLOC_016349	/	-0.53	4	6
miR399b	XLOC_020833	/	-0.53	5	3
miR399a	XLOC_008029	/	-0.51	5	6
miR399c-3p	XLOC_005358	/	-0.50	4.8	6
miR399f	XLOC_026270	/	-0.50	4.5	6

Note:

^a^Column3 describes functions of targeted genes of miR399

^b^PCC refers the expression correlation of miR399 and its target genes

^c^Target score is the target match score calculated by psRobot, and lower target score represents better base pair match between miRNA and targets

^d^The last column indicates the cluster that target genes or lncRNAs belonging to

## Discussion

With the advance of next-generation sequencing technology, many novel non-coding RNA transcripts have been found in different species. However, they were previously treated as transcriptional noise, because of their low expression levels and low evolutionary conservation [59, 60]. Recently, many lncRNAs have been recognized as important regulators of a variety of biological processes [61, 62]. Based on strand-specific RNA library construction protocols, a powerful tool in identifying NATs, nearly 10,000 lncRNAs have been annotated in the human genome [63–66]. However, although plants exhibit complicated biochemical, physiological, and developmental responses to cope with Pi starvation stress, a genome-wide characterization of known and novel lncRNAs involved in these responses is still lacking. In this study, we optimized experimental protocols with both poly(A) + and poly(A)– samples to capture genome-wide lncRNAs dynamically regulated under Pi starvation condition in both roots and shoots (Fig. 2). This is the first work to globally identify lncRNAs that respond to Pi starvation. Compared with previous genomic studies [21, 35, 37, 50, 51], we found many more Pi starvation-responsive protein-coding candidates with higher occurrences that in other studies (Additional file 1: Figure S10 and Additional file 4). However, in addition to the overlapped ones, we found that many differential expression genes we annotated were not reported in other studies and the repeatability of differential expression genes among other studies is also quite low (Additional file 1: Figure S10) [21, 35, 37, 50, 51]. It may be due to different growth stages and treatment conditions: we chose 10-day-old long-term Pi-starved seedlings grown on agar plates as plant material, which was the same as many previous studies [19, 20, 67]; while some of the other studies used more than 20-day-old plant with short-term Pi starvation treatment [35, 51] and others cultured the plants with hydroponic media [21, 50] or rockwool cubes [37]. Furthermore, different differential expression calculation tools used in different studies might also contribute to the differentially expressed candidates.

Based on the GO enrichment analyses of coding genes sharing the same expression pattern with the candidate lncRNAs, we found that many nuclear genome encoded PS-related genes were suppressed in both shoots and roots (Fig. 4c and Additional file 6) [35]. Although roots are heterotrophic organs and the expressions of PS genes were severely inhibited in roots compared with shoots (Additional file 1: Figure S15), there was still a basal level expression of PS genes in roots (Additional file 6). This might be because the seedlings were grown on agar plates and the roots were in the presence of light, which is a signal required for chlorophyll accumulation in roots [68]. We further showed that the expressions of genes involved in photosystem II and the redox chain were further down-regulated in both shoots and roots and the expressions of genes related to photosystem I were only further suppressed in roots under Pi starvation condition (Additional file 1: Figure S14). Based on these analyses, we speculated that Pi deprivation in shoots caused an adaptive strategy to reduce the expressions of PS-related genes in shoots. More severe suppression of the expression of photosynthetic genes in roots under Pi starvation condition may avoid excess production of reactive oxygen species (ROS) caused by aberrant PS activity, which can greatly damage cells [69]. Previous study demonstrated that suppression of PS gene expression is required for sustaining root growth under Pi deficiency [67]. Moreover, we also uncovered many candidate lncRNAs antisense to these PS genes that may play potential roles in regulating the suppression of PS genes. The function of these lncRNAs should be further investigated.

Moreover, the sensitive strand-specific RNA library construction protocol [70] enabled us to identify many novel lncRNAs transcribed from antisense strand. Interestingly, dozens of protein-coding genes, which were antisense to lncRNAs from cluster 3, were involved in cell wall organization processes (Fig. 4a, b). Meanwhile, the cluster 3 genes directly defined by expression pattern also showed high relevance with GO terms related to morphological changes, including cell wall organization and meristem development (Fig. 4b, c). These analyses indicated that the expression levels of genes involved in cell wall organization were tightly associated/correlated with their antisense lncRNAs during Pi starvation in shoots. Another antisense example was AT5G01595.1 and *AtFer1.* Their expression levels were both induced by Pi starvation, and their promoters both contained P1BS motifs, which indicated that they were both regulated by PHR1 during Pi starvation (Fig. 5a and Additional file 1: Figure S16). AtFer1 is a ferritin, which is stored in plastids to buffer free iron and maintain iron homeostasis [71, 72]. Previous study highlighted the biochemical, physiological, and molecular link between iron and Pi homeostasis [73, 74]. There is direct evidence that PHR1 can directly bind the promoter of *AtFer1* and up-regulate its expression under Pi starvation condition [52]. Taken together, our strand-specific RNA-Seq results revealed that antisense lncRNAs might have important roles in Pi starvation signaling pathways.

PHR1 and miRNA399 are two central transcriptional and post-transcriptional signal transducers in the regulatory network of Pi starvation responses. Thus, we conducted a genome-wide investigation of the P1BS motif in the promoter regions of lncRNAs and protein-coding transcripts. We found that the upstream regions of many differentially expressed lncRNA transcripts, as well as protein-coding transcripts, contained the P1BS motif (Table 1). In addition, most of these P1BS-associated transcripts were from clusters 1 and 2 (Fig. 5c and Additional file 1: Figure S17). These results indicated that PHR1 (and its homolog PHL1) mainly regulated the PSI genes in roots. This suggestion was supported by a previous split-root study [36]. However, many PSI genes in shoots only were less affected by PHR1 and their transcriptional regulators require further identification.

We also globally analyzed the regulatory network of miR399. We predicted 16 lncRNAs and 26 protein-coding genes as miR399 potential targets by integrating the sequence and expression relevance (Table 2). Here, we calculated correlation coefficient of miR399 and its target genes using 8 matched small RNA-seq and long RNA-seq datasets from different samples, including two replicates of P+ in roots and shoots, and two replicates of P– in roots and shoots. However, when we defined a gene or lncRNA was induced or repressed under Pi starvation condition, we just compared the expression in P+ and P– conditions. So the correlation of 8 samples could be inconsistent with the differential expression trend. A well-known example might explain this inconsistency is *IPS1*, it was found to be the target mimic of mi399 previously [27]. The expression level of *IPS1* was reported to be up-regulated under P– condition, although miR399 was induced as well. Our prediction recovered PHO2, the well-known target of miR399 [55, 75]. In addition, miR399 itself can be up-regulated by PHR1 [55]. Two lncRNAs, *IPS1* and *At4*, have been found to act as decoys of miR399 during Pi starvation [27, 76]. Integrating the above information, we proposed a Pi starvation signaling network illustrating the regulatory relationship among PHR1, miR399, PHO2, and their targets (Fig. 6). In addition to *IPS1*, we adapted a published method [56] to predict other potential target mimics of Pi deficiency regulated miRNAs (Additional file 2: Table S4). This method didn’t need an expression correlation, but it had more requirements on pairing rules (e.g., a three nucleotide bulge at the middle of miRNA binding site within target mimic’s sequence). In total, we have predicted 10 potential target mimics of Pi starvation responsive miRNAs (miR399, miR156 and miR169).

Furthermore, our study provided many candidate lncRNAs that could be simultaneously regulated by PHR1 and miR399 (Tables 1 and 2, and Additional file 7). Overall, we provided a set of research clues concerning the potential roles of the lncRNAs related to the signaling regulatory network under Pi starvation condition.

## Conclusions

In summary, we systematically identified thousands of novel poly(A) + and poly(A)– lncRNAs related to Pi starvation responses in Arabidopsis. We further characterized the known and novel lncRNAs and provided a rich candidate resource for future study. This work also revealed that many lncRNAs had potential roles in regulating mRNA levels of many protein-coding genes involved in Pi starvation responses. In addition, we proposed the coding–non-coding network of Pi starvation responses, based on the PHR1–miR399–PHO2 pathway. The next challenge in the field will be further functional studies to elucidate the specific molecular roles of these candidate lncRNAs and their association/interaction with other regulatory components involved in Pi starvation responses.

## Methods

### Plant materials and growth conditions

In this study, Arabidopsis wild type (Col-0) and *phr1* mutant (*Salk_067629*) plants were all of the Columbia ecotype background. The Pi-sufficient medium (P+) contained half-strength MS salts [77] with 1 % (w/v) sucrose and 1.2 % (w/v) agar (Sigma Cat. No. A1296). The formula of Pi-deficient medium (P–) was the same as for P+ medium except for replacing 1.25 mM KH_2_PO_4_ with 0.65 mM K_2_SO_4_. Seeds were surface sterilized with 20 % (v/v) bleach for 15 min and washed three times in sterile-distilled water. After that, seeds were sown on Petri plates containing P+ or P– medium and stratified at 4 °C for 2 d. The agar plates were placed vertically in a growth room at 22–24 °C and with a photoperiod of 16/8 h of light/dark. The light intensity was 100 μmol m^−2^ s^−1^. Two independent biological replicates of 10-day-old seedlings were collected and separated into roots and shoots for extraction of total RNA.

### Poly(A) + and poly(A)– RNA purification

We adapted a published RNA purification method to extract poly(A) + and poly(A)– RNA components from total RNA [78]. Firstly, we used DNase I (Promega) to treat total RNA and then incubated the DNA-free RNA with oligo(dT) magnetic beads (Oligotex mRNA Mini Kit, Qiagen). After the incubation, poly(A) + RNAs that were bound to the beads were isolated using centrifugation and resuspension. Poly(A)– RNAs, which were retained in the supernatant of incubation products, were processed with a Ribominus kit (RiboMinus™ Plant Kit for RNA-Seq, Invitrogen, A10838-08) to deplete ribosomal RNAs that account for the largest proportion of total RNA. Each reaction was performed twice to guarantee the purity of RNA components.

### Strand-specific RNA library construction and RNA sequencing

We used a dUTP-based method to construct strand-specific RNA libraries for poly(A) + and poly(A)– RNAs. After fragmentation of RNAs, they were reverse transcribed to cDNAs and then ligated to adaptors. Finally, fragments in the range of 300–500 nt were recovered using a gel extraction kit from PCR products. Then we sequenced samples with an Illumina Hiseq 2000/2500 platform to obtain paired end reads.

### Novel lncRNA identification pipeline

We used Tophat [79] to map reads to the TAIR10 [80] genome and used Cufflinks [81] to assemble transcripts based on 16 datasets of RNA-Seq samples. All the reads mapped to chloroplast and mitochondria genome were removed, and all protein-coding transcripts and lncRNAs that were used in this study were encoded by the nuclear genome. We assembled 60,027 transcripts that consisted of 31,139 protein-coding transcripts, 393 TAIR10 lncRNAs, 817 canonical ncRNAs, 862 pseudogenic transcripts, 3844 TE-related transcripts, and 22,972 new assembled transcripts. Other than protein-coding transcripts and TAIR10 lncRNAs, we followed the steps below to filter novel lncRNAs from the newly assembled transcripts: (i) 21,459 transcripts that overlapped with annotated coding-exons and non-coding RNA-exons in TAIR10 were filtered. Of these filtered transcripts, 21,434 had overlap with exons of protein-coding transcripts and TAIR10 lncRNA transcripts from the same strand. The other 24 transcripts had overlap with exons of TAIR10 lncRNA transcripts from the antisense strand; (ii) three transcripts of length < 200 nt were filtered; and (iii) coding potential for each transcript was calculated using CPC [82], and 298 transcripts with coding potential (CPC > 0) were filtered. Finally, 1212 transcripts that passed the above filter steps were retained as novel lncRNA transcripts. For the analyses below, the calculations were based on transcript level by default, except for the GO analysis of protein-coding genes.

### Classification of lncRNAs according to their genomic positions

We categorized the genomic positions of lncRNAs according to the overlap with genomic elements. If an lncRNA overlapped with a pseudogene or TE by more than one nucleotide, it was defined as a pseudogenic or TE-related lncRNA. If an lncRNA was located at the antisense strand of a protein-coding transcript, it was defined as antisense lncRNA. Other transcripts without any overlap or antisense relationship with annotated genes were classified as intergenic lncRNAs, parts of which were defined as *cis*-lncRNAs when the distance between lncRNAs and adjacent genes was ≤ 500 nt.

### Comparison with lncRNAs collected by PlncDB

We set two different criteria to overlap our defined lncRNAs with the lncRNAs in PlncDB: i. the overlapped length of a novel lncRNA with the collected lncRNA was more than 1 nt; ii. the ratio between the overlapped length and full length of a lncRNA was larger than 0.5. In the Additional file 3, we listed the overlapped ones based on criterion ii. PlncDB includes lncRNAs collected from five studies/resources, including EST datasets, two tilling array data sets (seedlings and seeds), RepTAS and RNA-seq studies [44–46].

### Classification of poly(A) + and poly(A)– lncRNAs

We classified lncRNAs into two groups: poly(A) + and poly(A)– lncRNAs. The criterion to define the type of lncRNAs was to compare the expression level of lncRNA of corresponding poly(A) + and poly(A)– samples. Firstly, we filtered low-expressed transcripts that had RPKM (Reads Per Kilobase of transcript per Million mapped reads) < 0.1 among 16 samples. For each transcript, we calculated a ratio of expression level (RPKM) of poly(A) + over expression level of poly(A)– sample. If the ratio for an lncRNA was ≥ 2, we defined it as a poly(A) + lncRNA; if the ratio was ≤ 0.5, we defined it as a poly(A)– lncRNA; and if the ratio was within 0.5–2, we classified them into a bimorphic group.

### Differential expressions under P+/P– conditions and transcript expression patterns in shoots and roots

We used eXpress [83] to measure the expression level of transcripts. The lncRNAs were assigned corresponding expression values according to their polyadenylation type: poly(A) + and bimorphic lncRNAs used expression values from poly(A) + samples; and expression levels of poly(A)– lncRNAs were based on poly(A)– samples. Then we used DESeq2 [84] to perform differential expression analysis. We calculated the fold-change of expression levels in Pi deficiency and Pi sufficiency and used *P-*values to filter the differentially expressed lncRNAs and protein-coding transcripts. We treated transcripts that had over two fold change with *P* < 0.05 as significantly differentially expressed; if transcripts only had over two fold change but without *P* < 0.05, we considered them not to be significantly expressed. Then we classified transcripts into six clusters according to their different levels of response in roots and shoots. Clusters 1 and 4 contained transcripts that were significantly induced or repressed in both roots and shoots. Clusters 2 and 5 included transcripts significantly induced or repressed only in roots. Clusters 3 and 4 consisted of transcripts significantly induced or repressed only in shoots.

### Validation of candidate lncRNAs with qRT-PCR

Total RNA was extracted with the Trizol reagent (Invitrogen) from 10-day-old seedlings. Of DNase-treated RNA, 2 μg was reverse transcribed in a 50-μL reaction using Takara MLV-Reverse transcriptase with random primer (Thermo) according to the manufacturer’s manual. cDNA was amplified using SYBR Premix Ex Taq (TaKaRa) on a Bio-Rad CFX96 real-time PCR detection system. Actin2 mRNA was used as an internal control. The genes and their primers are listed in Additional file 2: Table S3.

### GO enrichment and pathway enrichment analyses

We used DAVID [85] for the GO enrichment analyses of protein-coding genes among the six clusters. We used balloonplot of ggplot2 in the R statistical package [86] to visualize the result. We used MapMan [87] for pathway analysis of protein-coding genes. The input values for MapMan were the fold-changes of genes between P– and P+ conditions (Additional file 1: Figure S14), or the fold-changes of genes between roots and shoots under P+ condition (Additional file 1: Figure S15).

### P1BS motif search at the promoter regions and PHR1 target prediction

As coding sequences have been annotated very well for protein-coding genes, we treated the 2-kb upstream from the first ATG of a gene as its promoter. When lncRNAs lacked annotation, we set the upstream 2 kb from the start site of transcripts as their promoters. We used MEME [85] to generate a position weight matrix (PWM) for a given motif, based on the published P1BS motif sequence [12], and then processed with FIMO [53] to predict the motif enrichment at promoter regions of genes and lncRNAs (cutoff: *P* < 1e-3). As there were no available ChIP-Seq data for PHR1 in Arabidopsis, we also used other filter criteria to decrease the false positive rate of the target prediction based on motif search only. We required that these P1BS-motif enriched transcripts should respond to Pi starvation (significantly differentially expressed during P– treatment) and have chromatin accessibility (positive DNase value) at the promoter region (Additional file 7). We used the public DNase data [88] to measure the chromatin accessibility of the Arabidopsis whole genome.

### miRNA target prediction

We used psRobot [58] to predict targets of miRNAs. We set miRNA target score ≤ 5 as the cutoff for target prediction. We calculated Pearson’s correlation coefficient (PCC) to represent expression value of miRNAs and long transcripts. The cutoff for expression correlation was PCC < 0.5 [89].

## Abbreviations

ceRNA, competing endogenous RNA; GO, gene ontology; NAT, natural antisense transcript; NPA, poly(A)-; P–, Pi-deficient; P+, Pi-sufficient; PA, poly(A)+; R, root; S, shoot; TF, transcription factor

## Additional files

Additional file 1: Supporting **Figures S1–S22.** This file contains all supporting figures. (DOCX 11257 kb) Additional file 2: Supporting **Tables S1–S4.** This file contains all supporting tables. (DOCX 75 kb) Additional file 3: Coordinates of novel lncRNAs in RefFlat format. This file contains the detailed coordinates of each novel lncRNAs, including the start and end positions of exons. (XLSX 136 kb) Additional file 4: Differentially expressed protein-coding transcripts and lncRNAs under Pi starvation. This file contains all P– regulated protein-coding transcripts and lncRNAs. (XLSX 1710 kb) Additional file 5: Protein-coding genes and their (anti-)co-expressed antisense lncRNAs. This file contains all (anti-)co-expressed genes and their antisense lncRNAs pairs. (XLSX 69 kb) Additional file 6: Photosynthesis-related and P– repressed genes in roots and shoots. This file contains genes that are regulated by Pi starvation and involved in photosynthesis pathway. (XLSX 33 kb) Additional file 7: LncRNAs and protein-coding genes with P1BS motif at promoters. This files contains lncRNAs and protein-coding genes whose promoter regions contain P1BS motif. (XLSX 107 kb)
