# Supplementary material for: Systematic characterization of novel lncRNAs responding to phosphate starvation in Arabidopsis thaliana
Source: BMC Genomics. 2016 Aug 18;17:655. doi: 10.1186/s12864-016-2929-2 (PMC4991007; doi:10.1186/s12864-016-2929-2)
Supplement: Additional file 1: — Supporting Figures S1–S22. This file contains all supporting figures. (DOCX 11257 kb) [file 12864_2016_2929_MOESM1_ESM.docx]

**Supporting Information**

Supporting Figures 2

Figure S1. Morphological appearance of *Arabidopsis* seedling grown for 10 d on Pi-sufficient or -deficient media. Bar = 0.5 cm. 2

Figure S2. Proportion of all assembled transcripts. 3

Figure S3. Polyadenylation proportion of lncRNAs. 4

Figure S4. Genomic features of poly(A)+ and poly(A)– lncRNAs. 5

Figure S5. Saturation plot of protein-coding transcripts, TAIR10 lncRNAs and novel lncRNAs. 6

Figure S6. Epigenetic signatures of lncRNAs around transcription start site (TSS). 7

Figure S7. Sequence conservation (phastCons score) of lncRNAs. 8

Figure S8. Sequence and structure conservation of novel lncRNAs. 9

Figure S9. Validation of expression levels of bimorphic lncRNAs in poly(A)+ and poly(A)– samples. 10

Figure S10. Comparative analysis of P– responsive protein-coding genes that identified via RNA-Seq and array. 11

Figure S11. More candidate P– responsive lncRNAs were validated in roots and shoots. 12

Figure S12. Polyadenylation for the lncRNAs in different clusters. 13

Figure S13. Differential alternative splicing events of lncRNAs among clusters. 14

Figure S14. Repressed photosynthetic genes in roots and shoots under P– condition during photosynthesis. 15

Figure S15. Repressed photosynthetic genes in roots under P+ condition during photosynthesis. 16

Figure S16. qRT-PCR validation of *AtFer1* and AT5G01595 in *phr1* mutant. 17

Figure S17. Enrichment of P1BS motif at the promoters of protein-coding transcripts and lncRNAs among clusters. 18

Figure S18. Correlation between P1BS motif numbers and fold-change of expression levels for protein coding transcripts. 19

Figure S19. Position preference of P1BS motif. 20

Figure S20. Protein-coding genes regulated by PHR1 were less induced in *phr1* and *phr1phl1* mutant. 21

Figure S21. qRT-PCR validation of protein-coding marker genes in *phr1* mutant. 22

Figure S22. miRNAs response to phosphate starvation. 23

References 24

# Supporting Figures

## Figure S1. Morphological appearance of *Arabidopsis* seedling grown for 10 d on Pi-sufficient or -deficient media. Bar = 0.5 cm.


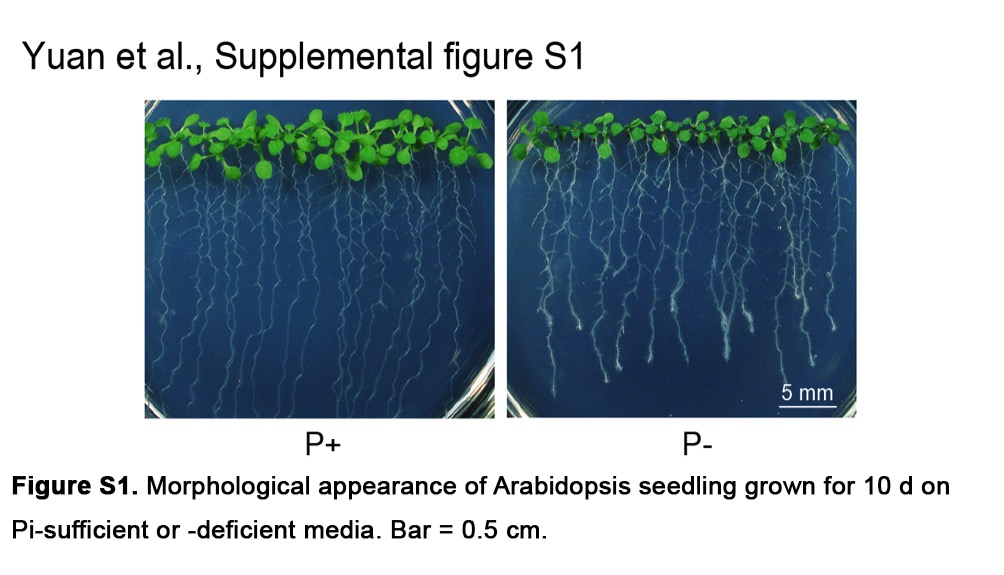


## Figure S2. Proportion of all assembled transcripts.

#
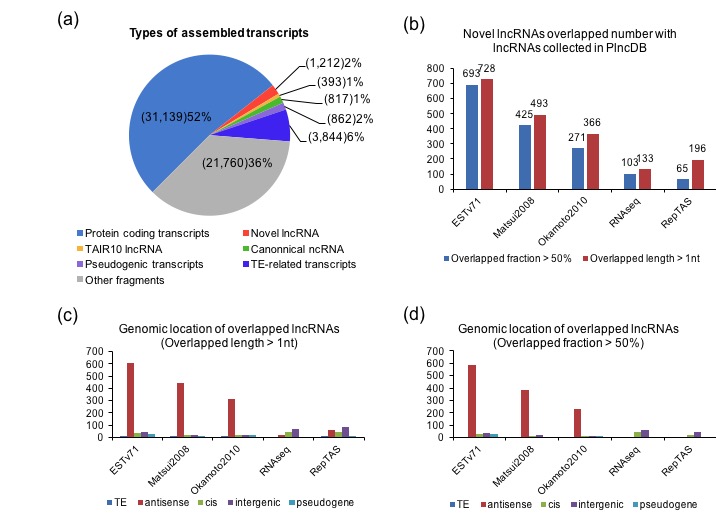


**(a)** Subsequently, 60,027 transcripts were assembled in total, which consisted of 31,139 protein-coding transcripts, 393 TAIR10 lncRNAs, 817 canonical ncRNAs, 862 pseudogenic transcripts, 3,844 TE-related transcripts and 22,972 new assembly transcripts. Among these transcripts, mostly were protein-coding transcripts, and lncRNAs account for a small fraction of all assembled transcripts. Almost third of assembled transcripts (labeled as other fragments) had been filtered because of overlaps with annotations of TAIR10 or high coding potentials. During the novel lncRNAs filter steps: firstly, we filtered 21,459 transcripts that overlapped with coding-exons and noncoding RNA-exons annotated in TAIR10; secondly, we filtered 3 transcripts that were less than 200nt; thirdly, we filtered 298 transcripts with high coding-potential (CPC>0). Finally, we obtained 1,212 novel lncRNA transcripts.

**(b)** We compared our defined 1,212 novel lncRNAs with those lncRNAs collected by PlncDB. These lncRNAs were collected from previous five studies. We set two different criteria to overlap our defined lncRNAs with those lncRNAs that PlncDB collected. We found many of our defined lncRNAs were overlapped with NATs defined by EST and other two tilling array studies. And a small fraction of lncRNAs was overlapped with lincRNAs that defined by the RNAseq and RepTAS studies.

**(c)-(d)** We compared the genomic location of overlapped lncRNAs to find that majority of these lncRNAs that overlapped with NATs were antisense lncRNAs, which was an expected result.

## Figure S3. Polyadenylation proportion of lncRNAs.

**(a)** Polyadenylation proportion of TAIR10 annotated lncRNAs.

**(b)** Polyadenylation proportion of novel lncRNAs.

Over 70% of TAIR10 annotated and novel lncRNAs were defined as poly(A)+ transcripts. Interestingly, more lncRNAs were defined as poly(A)– transcripts under P– condition than under P+ condition. Moreover, more lncRNAs were exchanged from poly(A)+ group to bimorphic group because of their decreased abundances in poly(A)+ samples or increased expression levels in poly(A)– samples (data not shown).

## Figure S4. Genomic features of poly(A)+ and poly(A)– lncRNAs.

**(a)** LncRNAs have shorter transcript length than protein-coding transcripts.

**(b)** LncRNAs have shorter exon length than protein-coding transcripts.

**(c)** Poly(A)– transcripts of lncRNAs tended to have lower GC content than other types.

We compared the TAIR10 annotated and novel lncRNAs in different levels, such as transcript length, exon length, and GC content. Novel lncRNAs had longer exon length than protein-coding transcripts due to sufficient sequencing depth to achieve almost real full length of exons (Table S2). When compared poly(A)+ and poly(A)– lncRNAs within a certain type, we found that poly(A)+ lncRNAs showed longer transcript length and more exon number than poly(A)– transcripts (Figure S3A, S3B, 2B, Table S2). Interestingly, poly(A)– transcripts harvested in longer exon length than poly(A)+ transcripts (Figure S4C, Table S2). Poly(A)– lncRNAs tended to have lower GC content than other types, especially for annotated poly(A)– transcripts (Figure S4D, Table S2) [1].

## Figure S5. Saturation plot of protein-coding transcripts, TAIR10 lncRNAs and novel lncRNAs.

**(a)** Saturation plot of protein-coding transcripts**.**

**(b)** Saturation plot of TAIR10 annotated lncRNAs.

**(c)** Saturation plot of novel lncRNAs.

**Datasets**: Number of RNA-seq data used. **Ratio**: the proportion that the number of transcripts expressed based on a certain number of datasets relative to the number of total transcripts expressed based on all 16 RNA-seq datasets. **Expression cutoff**: protein-coding transcripts and lncRNAs that mapped with at least 5 RNA-seq reads were defined as expressed transcripts.

From the saturation plot of protein-coding genes and lncRNAs, we noticed that detected ratio of TAIR10 annotated and novel lncRNAs were dependent on the number of RNA-seq datasets used. Along with more RNA-seq data added, more lncRNAs could be assembled and the ratio tended to be stable until about 10 datasets were used. That is to say, detection of lncRNAs relied on the amount and abundance of datasets. Whereas, due to the higher expression levels of protein-coding transcripts, they could be detected only based on a few of datasets so that they only needed about 6 datasets to reach the complete assembled ratio.

## Figure S6. Epigenetic signatures of lncRNAs around transcription start site (TSS).

**(a)-(c)** H3K4me2, H3K4me3 and H3K9Ac signal were enriched around the transcription start site (TSS).

**(d)** DNase peaks were enriched around TSS.

To determine whether the novel lncRNAs were independent transcripts rather than degradation products or extensions of coding genes, we combined public ChIP-Seq data for epigenetic signatures that was associated with transcriptional activity, including H3K4me2, H3K9Ac and H3K4me3. We observed enrichment of histone modifications around transcription start sites (TSS) for both coding and non-coding transcripts. Protein-coding transcripts had the highest enrichment, following the TAIR10 lncRNAs and novel lncRNAs. LncRNAs showed much higher enrichment than shuffled transcripts to indicate they were actively transcribed.

Moreover, we used DNase data to measure chromatin accessibilities around TSS of coding and non-coding transcripts. We found that DNase peaks of all kinds of transcripts were remarkably enriched around TSS when compared with shuffled ones.

## Figure S7. Sequence conservation (phastCons score) of lncRNAs.

We further analyzed the conservation levels of lncRNAs. We separated exons and introns of each kind of transcripts to assign the highest phastcons score within the region. Two trends were observed that coding transcripts were more conserved than noncoding ones and exons always showed higher phastCons score than introns of a certain type of transcript. Interestingly, the exons of novel lncRNAs were more conserved than exons of TAIR10 lncRNAs and introns of protein-coding transcripts.

## Figure S8. Sequence and structure conservation of novel lncRNAs.

**Rfam:** lncRNA have conserved structure annotated in Rfam. **Blast:** lncRNA have conserved sequence with canonical ncRNAs annotated in TAIR10.

We used Blast to align sequences of novel lncRNA transcripts to the TAIR10 annotated canonical ncRNAs, including rRNA, tRNA, miRNA, snRNA and snoRNA. We found 20 novel lncRNAs were sequence conserved with the annotated canonical ncRNAs. Besides, we used INFERNAL[2] to scan *Arabidopsis* genome to search conserved structures of novel lncRNAs that were homologous with annotations in Rfam. We found 10 lncRNAs, among which 9 lncRNAs that were both sequence and structure conserved. Since lncRNAs having conserved structures or sequences with canonical ncRNAs have been reported to be functional in biological processes [3, 4], we kept these 21 conserved lncRNAs for further study.

## Figure S9. Validation of expression levels of bimorphic lncRNAs in poly(A)+ and poly(A)– samples.

**(a)** Validation of TAIR10 bimorphic lncRNAs.

**(b)** Validation of novel bimorphic lncRNAs.

**PA:** lncRNAs enriched in poly(A)+ samples; **NPA:** lncRNAs enriched in poly(A)– samples. Bimorphic lncRNAs didn’t show distinguishable expression differences between poly(A)+ and poly(A)– samples so that they could be defined as neither poly(A)+ lncRNAs nor poly(A)– lncRNAs. Bimorphic lncRNAs might generated from poly(A)+ transcripts and then processed to shorten or remove their poly(A) tails, which could be supported by evidences that there were a quarter of genes had a short poly(A) tail of less than 30 nucleotides[5, 6]. Moreover, about 24% lncRNAs had proven they may lack a classic poly(A) tail[7, 8]. These studies threw light on the existences of bimorphic and poly(A)– lncRNAs.

## Figure S10. Comparative analysis of P– responsive protein-coding genes that identified via RNA-Seq and array.

**(a)** Comparison of P– responsive genes that identified by RNA-seq and microarray.

**(b)** Comparison of P– responsive genes that identified by RNA-seq and microarray in roots and shoots, respectively.

Venn diagrams showed overlap number of P– regulated genes from different studies. Because of the deep sequencing depth of RNA-seq data, more P– responsive protein-coding genes and lncRNAs were detected based on our datasets compared with previous array data [9-13]. All of these studies had identified hundreds of P– induced or repressed protein-coding genes, and some studies[9, 13] separated roots and shoots as well. Taken the experimental variations and batch effects into consideration, we found that candidate P– regulated genes generated from RNA-seq were well reproduced with array data.

## Figure S11. More candidate P– responsive lncRNAs were validated in roots and shoots.


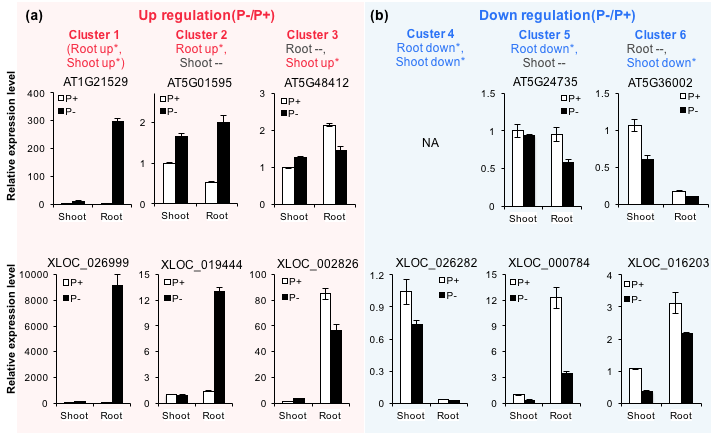


**(a)** Validation of P– induced TAIR10 lncRNAs and novel lncRNAs.

**(b)** Validation of P– repressed TAIR10 lncRNAs and novel lncRNAs.

**P+**: relative expression levels of lncRNAs under Pi sufficient condition (labeled as white bar). **P–:** relative expression levels of lncRNAs under Pi deficient condition (labeled as black bar). The light pink and link blue background represented lncRNAs that were induced or repressed under Pi starvation conditions, respectively.

In addition to the main figure, we show more candidate lncRNAs from the six clusters, which were confirmed with qRT-PCR. Their relative expression levels were consistent with RNA-seq data. The y-axis value referred to relative expression levels that measured by qRT-PCR.

## Figure S12. Polyadenylation for the lncRNAs in different clusters.

##

**(a)** Polyadenylation of TAIR10 differentially expressed lncRNAs of 5 clusters.

**(b)** Polyadenylation of novel differentially expressed lncRNAs of 6 clusters.

Based on the previous results of poly(A)+ and poly(A)– classification (Figure 2A), we counted polyA(+) and poly(A)– lncRNAs for each cluster. The TAIR10 annotated lncRNAs of cluster 1, 2 and 5 were entirely classified into poly(A)+ group. Meanwhile, there were a comparable number of poly(A)– lncRNAs in cluster 2, 3, 5 and 6 for the novel lncRNAs. This was expected because only our study had sequenced the nonpolyA RNA libraries.

## Figure S13. Differential alternative splicing events of lncRNAs among clusters.

**(a)** Differentially alternative splicing of TAIR10 lncRNAs of 5 clusters.

**(b)** Differentially alternative splicing of novel lncRNAs of 6 clusters

**SE**: Skipped exon; **RI**: Retained intron; **MXE**: Mutually exclusive exons; **A5SS**: Alternative 5’ splice site; **A3SS**: Alternative 3’ splice site.

Differentially alternative splicing events happened for both TAIR10 and novel lncRNAs under P– conditions. It showed that differentially splicing events were mainly enriched in lncRNAs of cluster 6. A couple of novel lncRNAs in cluster 5 were detected A3SS type alternative splicing events. Main patterns of alternative splicing were retained intron (RI), alternative 3’ splice site (A3SS) and skipped exon (SE).

## Figure S14. Repressed photosynthetic genes in roots and shoots under P– condition during photosynthesis.

Differentially expressed genes and lncRNAs of clusters 5 and 6 that were involved in light reactions of photosynthesis. P– repressed genes in roots and shoots and their antisense lncRNAs were labeled. From these photosynthetic genes and lncRNAs, the response patterns of light reactions in roots and shoots were found to differ, especially in photosystem I.

## Figure S15. Repressed photosynthetic genes in roots under P+ condition during photosynthesis.

It is well known that photosynthesis reactions in roots are severely suppressed because of the lack of chloroplast[9]. Thus, we compared expression levels of photosynthetic genes in roots and shoots under P+ condition. It turned out to be that a number of genes were repressed in roots during light reactions of photosynthesis under normal condition compared with in shoots, which indicated that the suppression of photosynthesis process in roots had happened under normal condition. Combined this finding with the results shown in Figure 4d, we observed that a proportion of photosynthesis-suppressed genes in roots were even heavier repressed under P– condition, which was consistent with previous studies[9-11].

## Figure S16. qRT-PCR validation of *AtFer1* and AT5G01595 in *phr1* mutant.

qRT-PCR validation of *AtFer1* and AT5G01595 in *phr1* mutant. Both of *AtFer1* and AT5G01595 had P1BS motif at their promoter regions, and they were less induced in *phr1* mutant.

## Figure S17. Enrichment of P1BS motif at the promoters of protein-coding transcripts and lncRNAs among clusters.

**(a)** P1BS motif enrichment of differentially expressed genes of 6 clusters. Compared motif enriched ratio of all differentially expressed genes of 6 clusters, we found that P1BS were significantly enriched at promoters of genes in cluster 1 and 2, approximately to 60% and 45%. **All:** We set all protein-coding transcripts as control. The difference in ratios is tested using χ² test: **a** compared with **b**, *P-value*<0.01; no significant difference within **a** and **b.**

**(b)-(c)** Average P1BS content at promoters of motif enriched genes or lncRNAs in cluster 1-3. **P– regulated coding transcripts/lncRNAs** referred to all P– responsive protein-coding transcripts/lncRNAs among 6 clusters. We found more P1BS motifs at the promoters of lncRNAs belonging to cluster 1 and 2 compared with all phosphate starvation regulated lncRNAs. The same trend could be observed for protein-coding genes. Motif-enriched lncRNAs of cluster 2 had almost 2 P1BS motifs at promoters on average, as well as the protein-coding genes with P1BS motif in cluster 1, which provided more accessibility for PHR1 to target.

## Figure S18. Correlation between P1BS motif numbers and fold-change of expression levels for protein coding transcripts.

**(a)** Correlation between P1BS content and fold of induction in Pi starvation in roots.

**(b)** Correlation between P1BS content and fold of induction in Pi starvation in shoots.

Along with the number of P1BS motif at promoters of protein-coding genes among cluster 1-3 was increasing, their fold change of expression level induced by Pi starvation was raised. It suggested that inducibility of PHR1 had a correlation with the P1BS motif number at promoter regions. The header numbers in the gray box standard for motif number.

## Figure S19. Position preference of P1BS motif.

**(a)** Position preference of P1BS motif in promoters of lncRNAs.

**(b)** Position preference of P1BS motif in promoters of protein coding genes.

**P– regulated lncRNAs/coding transcripts** referred to all P– responsive lncRNAs or protein-coding transcripts among 6 clusters. We did further analysis to test position distribution of P1BS motif of lncRNAs and genes among the up-regulated transcripts (cluster 1-3). We separated the promoter region into two regions, proximal and distal. The proximal region was the 1kb region that next to the gene body, and distal region referred to the 1kb that located further to the gene body. Thus, we measured the motif enrichment of the two regions to see the position preference of P1BS. It was interesting to find that most P1BS motif of lncRNAs from cluster 1 tended to locate in proximal promoter region. The position preference of P1BS could also be observed in protein-coding genes.

## Figure S20. Protein-coding genes regulated by PHR1 were less induced in *phr1* and *phr1phl1* mutant.

**(a)** All of these genes were significantly induced by phosphate starvation in roots and shoot, called P– starvation marker genes. Furthermore, public array data of *phr1* and *phr1phl1* mutant were combined to measure their fold change of expression level induced by Pi starvation. It was obviously that the fold inductions of all marker genes were reduced in *phr1* and *phr1phl1* mutant.

**(b)** Fold inductions of protein-coding genes in cluster1 were lower in *phr1* and *phr1phl1* mutant. Protein-coding genes in cluster 1 were the set of genes that induced most significantly under Pi starvation and had more possibilities to be regulated by PHR1. There was a similar pattern that their fold change of expression level induced by Pi deficient in *phr1* and *phr1phl1* mutant were decreased compared with the fold change in Col-0.

**(c)** Protein-coding genes that without P1BS motif at promoter and without fold induction under Pi deficient condition didn’t show apparent expressional change between Col-0 and mutants. Genes that without P1BS motif in promoter and induction by Pi starvation were chosen as negative control, and they weren’t detected any apparent expressional change between Col-0 and mutants.

## Figure S21. qRT-PCR validation of protein-coding marker genes in *phr1* mutant.

qRT-PCR validation of Pi starvation induced (PSI) genes in *phr1* mutant. We randomly picked some marker genes with P1BS motif at promoters to validate their expression level in *phr1* mutant using qPCR. Marker genes showed lower fold changes in *phr1* mutant that induced by Pi starvation compared with the value in Col-0.

## Figure S22. miRNAs response to phosphate starvation.

**(a)** miRNAs regulated in roots under P– condition.

**(b)** miRNAs regulated in shoots under P– condition.

Several miRNAs revealed to be differentially expressed in roots and shoots under Pi starvation. 13 and 11 miRNAs were significantly induced in roots and shoots, respectively.

# References

1. Tanne A, Muniz LR, Puzio-Kuter A, Leonova KI, Gudkov AV, Ting DT, Monasson R, Cocco S, Levine AJ, Bhardwaj N *et al*: **Distinguishing the immunostimulatory properties of noncoding RNAs expressed in cancer cells**. *Proceedings of the National Academy of Sciences of the United States of America* 2015, **112**(49):15154-15159.

2. Nawrocki EP, Kolbe DL, Eddy SR: **Infernal 1.0: inference of RNA alignments**. *Bioinformatics* 2009, **25**(10):1335-1337.

3. Wilusz JE, Freier SM, Spector DL: **3' end processing of a long nuclear-retained noncoding RNA yields a tRNA-like cytoplasmic RNA**. *Cell* 2008, **135**(5):919-932.

4. Hsieh LC, Lin SI, Shih AC, Chen JW, Lin WY, Tseng CY, Li WH, Chiou TJ: **Uncovering small RNA-mediated responses to phosphate deficiency in Arabidopsis by deep sequencing**. *Plant physiology* 2009, **151**(4):2120-2132.

5. Gu H, Das Gupta J, Schoenberg DR: **The poly(A)-limiting element is a conserved cis-acting sequence that regulates poly(A) tail length on nuclear pre-mRNAs**. *Proceedings of the National Academy of Sciences of the United States of America* 1999, **96**(16):8943-8948.

6. Meijer HA, Bushell M, Hill K, Gant TW, Willis AE, Jones P, de Moor CH: **A novel method for poly(A) fractionation reveals a large population of mRNAs with a short poly(A) tail in mammalian cells**. *Nucleic acids research* 2007, **35**(19):e132.

7. Wu Q, Kim YC, Lu J, Xuan Z, Chen J, Zheng Y, Zhou T, Zhang MQ, Wu CI, Wang SM: **Poly A- transcripts expressed in HeLa cells**. *PloS one* 2008, **3**(7):e2803.

8. Cui P, Lin Q, Ding F, Xin C, Gong W, Zhang L, Geng J, Zhang B, Yu X, Yang J *et al*: **A comparison between ribo-minus RNA-sequencing and polyA-selected RNA-sequencing**. *Genomics* 2010, **96**(5):259-265.

9. Wu P, Ma L, Hou X, Wang M, Wu Y, Liu F, Deng XW: **Phosphate starvation triggers distinct alterations of genome expression in Arabidopsis roots and leaves**. *Plant physiology* 2003, **132**(3):1260-1271.

10. Misson J, Raghothama KG, Jain A, Jouhet J, Block MA, Bligny R, Ortet P, Creff A, Somerville S, Rolland N *et al*: **A genome-wide transcriptional analysis using Arabidopsis thaliana Affymetrix gene chips determined plant responses to phosphate deprivation**. *Proceedings of the National Academy of Sciences of the United States of America* 2005, **102**(33):11934-11939.

11. Morcuende R, Bari R, Gibon Y, Zheng W, Pant BD, Blasing O, Usadel B, Czechowski T, Udvardi MK, Stitt M *et al*: **Genome-wide reprogramming of metabolism and regulatory networks of Arabidopsis in response to phosphorus**. *Plant Cell Environ* 2007, **30**(1):85-112.

12. Muller R, Morant M, Jarmer H, Nilsson L, Nielsen TH: **Genome-wide analysis of the Arabidopsis leaf transcriptome reveals interaction of phosphate and sugar metabolism**. *Plant physiology* 2007, **143**(1):156-171.

13. Woo J, MacPherson CR, Liu J, Wang H, Kiba T, Hannah MA, Wang XJ, Bajic VB, Chua NH: **The response and recovery of the Arabidopsis thaliana transcriptome to phosphate starvation**. *BMC plant biology* 2012, **12**:62.
