# Supplementary material for: Systematic characterization of novel lncRNAs responding to phosphate starvation in Arabidopsis thaliana
Source: BMC Genomics. 2016 Aug 18;17:655. doi: 10.1186/s12864-016-2929-2 (PMC4991007; doi:10.1186/s12864-016-2929-2)
Supplement: Additional file 2: — Supporting Tables S1–S4. This file contains all supporting tables. (DOCX 75 kb) [file 12864_2016_2929_MOESM2_ESM.docx]

**Supporting Information**

Supporting Tables 2

Table S1. Mapping ratios of samples 2

Table S2. Summary of characteristics of lncRNAs 3

Table S3. Sequences of the primers used for quantitative real-time PCR. 4

Table S4. Correlation between P– regulated miRNAs and potential target mimics 5

References 6

# Supporting Tables

## Table S1. Mapping ratios of samples

| **Ecotype** | **Tissue** | **Condition** | **Library Type** | **Total reads** | **Mapped reads** | **Mapping reads ratio** | **Mapping chrPt and chrMt ratio^1^** |
| --- | --- | --- | --- | --- | --- | --- | --- |
| Col-0 | Root | P– | Poly(A)– | 31,438,216 | 29,131,388 | 92.70% | 14.70% |
| Col-0 | Root | P– | Poly(A)– | 23,641,042 | 22,508,820 | 95.20% | 17.10% |
| Col-0 | Root | P– | Poly(A)+ | 32,339,258 | 30,362,997 | 93.90% | 2.30% |
| Col-0 | Root | P– | Poly(A)+ | 26,521,464 | 25,040,530 | 94.40% | 0.90% |
| Col-0 | Shoot | P– | Poly(A)– | 18,181,630 | 17,326,819 | 95.30% | 70.90% |
| Col-0 | Shoot | P– | Poly(A)– | 15,634,394 | 14,787,050 | 94.60% | 61.90% |
| Col-0 | Shoot | P– | Poly(A)+ | 24,620,434 | 23,186,351 | 94.20% | 7.80% |
| Col-0 | Shoot | P– | Poly(A)+ | 23,482,546 | 21,650,500 | 92.20% | 5.60% |
| Col-0 | Root | P+ | Poly(A)– | 37,998,498 | 35,401,640 | 93.20% | 5.70% |
| Col-0 | Root | P+ | Poly(A)– | 21,813,768 | 20,744,633 | 95.10% | 15.70% |
| Col-0 | Root | P+ | Poly(A)+ | 42,398,304 | 38,558,711 | 90.90% | 0.30% |
| Col-0 | Root | P+ | Poly(A)+ | 27,145,560 | 25,869,361 | 95.30% | 0.70% |
| Col-0 | Shoot | P+ | Poly(A)– | 25,729,416 | 24,574,889 | 95.50% | 70.30% |
| Col-0 | Shoot | P+ | Poly(A)– | 17,540,162 | 16,641,362 | 94.90% | 66.20% |
| Col-0 | Shoot | P+ | Poly(A)+ | 24,137,118 | 23,038,009 | 95.40% | 3.80% |
| Col-0 | Shoot | P+ | Poly(A)+ | 32,508,476 | 30,895,902 | 95.00% | 2.20% |

^1^Ratio of reads mapped to chloroplast and mitochondria genome of *Arabidopsis* genome.

We found that 60-70% of the reads from shoot’s poly(A)– RNA samples were mapped to chloroplast and mitochondria genomes of *Arabidopsis*, which was consistent with previous studies showing the majority of poly(A)– RNAs in shoot and leaves were from chloroplast and mitochondria (Gagliardi *et al.* 2001, Schuster *et al.* 1999).

## Table S2. Summary of characteristics of lncRNAs

| **Type** | | **Protein-coding transcripts** | **TAIR10 poly(A)+ lncRNAs** | **TAIR10 poly(A)**– **lncRNAs** | **Novel poly(A)+ lncRNAs** | **Novel poly(A)**– **lncRNAs** |
| --- | --- | --- | --- | --- | --- | --- |
| **Exon length/nt** | **Min.** | 1 | 3 | 31 | 1 | 10 |
|  | **Median** | 160 | 274 | 212.5 | 487 | 643 |
|  | **Max.** | 7761 | 2614 | 2742 | 6385 | 4582 |
| **Exon Number** | **Min.** | 1 | 1 | 1 | 1 | 1 |
|  | **Median** | 4 | 2 | 2 | 2 | 1 |
|  | **Max.** | 79 | 10 | 5 | 17 | 4 |
| **Transcript length/nt** | **Min.** | 75 | 204 | 126 | 207 | 278 |
|  | **Median** | 1374 | 878 | 563.5 | 1126 | 963 |
|  | **Max.** | 16347 | 3426 | 5159 | 6385 | 4818 |
| **GC content** | **Min.** | 0.2481 | 0.2823 | 0.3149 | 0.2742 | 3031 |
|  | **Median** | 0.4214 | 0.4104 | 0.377 | 0.4093 | 0.4102 |
|  | **Max.** | 0.6901 | 0.563 | 0.5856 | 0.5515 | 0.5504 |

Detailed number of exon length, exon number, transcript length and GC content for protein-coding transcripts, TAIR10 annotated poly(A)+/– lncRNAs, novel poly(A)+/– lncRNAs were listed in Table S3. LncRNAs revealed longer exon length than protein-coding transcripts, especially novel lncRNAs. Protein-coding transcripts exhibited the most exon number and the longest transcript length of these types. Poly(A)+ lncRNAs had longer transcript than poly(A)– lncRNAs did and poly(A)– lncRNAs showed lower GC content than other types.

## Table S3. Sequences of the primers used for quantitative real-time PCR.

| **Gene/lncRNA ID** | **Forward Primer** | **Reverse Primer** |
| --- | --- | --- |
| AT1G10682 | CAGAGGAACCTCAGTTTGGAAAAT | TGCCGTTTTGTCTCCTCCTT |
| AT2G14878 | GGAACCGGTGATTGTTCATCA | CTCCGTTGGCTTCAAGCAA |
| AT2G19572 | TTATACGCCGCCGATTCC | TTATGTCCATCGGGAGTTTCG |
| AT2G46192 | TCGCCGTTTCGGCAACT | TTCATCGCATCGCTCGATAA |
| AT5G06165 | AGGTCCTCTCTCGCTGACTCTTC | TGAAAATCCTCAAACGCAAAGAT |
| AT3G59068 | CGGATGGTGGTAGGAATGAAA | TCATGATCCCCTCAGTTTTTGTT |
| XLOC_000212 | ATCCTCACGTGCCTCTGAGAA | TGTACGCCAAACGTGCTTTC |
| XLOC_000344 | ATACATAGGAAAATACAAAGCTGGGAAA | GGAATAAAGACGTGACCGTTGTG |
| XLOC_000354 | AACGTCTCTCTCACTTGGCTTCTC | GAGGAAGGTTTGATGAGGAGCAT |
| XLOC_022857 | TCTGAGCTGCCCAGTCCAA | TCCGACTCTAGAACCTGCATGA |
| XLOC_004039 | AAATTGGTACCCCGAGTGAGAA | CACTCAGTGGAAGCCAACCAT |
| XLOC_025632 | AGCAGTGGGTCGAGCTATGTAAC | CCGTGGGCCGATCCA |
| AT1G50055 | CGTCAAGCTCTGCAACTACGAT | AACAGCGGAACCTGATCTAAGAA |
| AT2G24545 | GAGGAGACGAGATTTTGGTGAAA | GCAATCCCTTGACCTTGCAT |
| AT4G02005 | AAAACATGAGCCTTTGATACCAAAT | TGTGGAAGATTGAAGGAAAGCTAA |
| XLOC_007967 | CGTAACGCCTCAATCGTCTTC | GGAGAACAAGCCGTCAACGT |
| XLOC_019375 | CCGAAGAACTTAGCGACGAAGA | ATCCTCCGTCCATGTATTCAATG |
| XLOC_022497 | GACGGTGTTGCCTTGGGATA | GCACTCCATGGCCAGATGTT |
| AT3G12502 | GTGGGTCCAGGAGGAGTACCA | TGCGGTAACACCGAACCATA |
| AT5G06865 | CGATCCGGTTAAAGTCGATGTT | CCCTGACCTTCGCCTATCAC |
| AT1G67328 | GAAAATATCGCCGCCATCCT | ACCGAGTGGGATCTGATCATG |
| AT5G24735 | AAAAAGCGATTCATGGCAAGA | TTGATGCTGGAAACGAACCA |
| AT5G36002 | TCCTGGGTAGTCACATGACGAA | CATGTGGAGGAAACAACCTTGA |
| AT1G21529 | CGGTGAGCGTGGATCGA | CTGGCACCAATACCCATTCAT |
| AT5G01595 | CGAGAGAAACCGACGGAGAA | GTCTTTCACCGCCGCTAATC |
| AT5G48412 | CACGTGTCTTCAGCACCTAACC | CCGCATCAGGAAGAAAGAAGAA |
| AT3G48115 | GCGAGGATTTCTTACCGGAAT | CCCACGCCTGATGAGTAATCA |
| AT1G06265 | ACACCGGAGGAGTAGAGCTGAA | CAAATCGCTGCAGCTCAACA |
| XLOC_030250 | ACCATAAGTGCTTTCGGACAATC | TATTGTGTGCTCGGGTATAAGTTTG |
| XLOC_016379 | CCACGTCAGAGGATACATAATTATCAA | TGTGTAAAGGTTGCTTGCAATCA |
| XLOC_013570 | ATTGGTGATGGCAGCACTCA | AGACGGCTGATTCCACCAAA |
| XLOC_016321 | TCTTTAGCTCCGCTCCATCAA | CGTTCCCGGAGAAGGATTTC |
| XLOC_003425 | GACAAAAGAACAAGAGGGCGATA | TTTCTCACGTTTTGGTGATTGG |
| XLOC_028295 | GTCCCCTGTTTCGAGGCATT | TCTTACCGTGGCTTTCAACAAG |
| XLOC_026999 | TGATTGGTAGGCATCATGTTCAA | GGCCAACTCCACGTTCGT |
| XLOC_019444 | TAATCGCCGGAGCTCAGAAT | CCATGGCCAATCATCTCAACT |
| XLOC_002826 | GCAAAACAAGTGAAACAAATTGAGA | TGTATGATGCCATCTTGGATGTG |
| XLOC_000784 | TCCAGAAGCAACATGGATCCT | TCTGCCAGAAACGCCAACA |
| XLOC_026282 | GTTCCGGACCTAGTGCCAAA | TTGTCTCCATCGCGGATTATC |
| XLOC_016203 | CCGTTTCCAACCCAAAATCA | CATGTCTGGCTACCGCATTG |
| AT2G14878 | GGAACCGGTGATTGTTCATCA | CTCCGTTGGCTTCAAGCAA |
| AT5G48412 | CACGTGTCTTCAGCACCTAACC | CCGCATCAGGAAGAAAGAAGAA |
| AT3G17185 | GAGAGAGAAGAGCTCCCATGGA | CATCACGGAGGTTATGTTTTCCT |
| AT5G53048 | CCAGCCGAGCGGATTAGTT | TACGGGTTTGATGGCCAAAT |
| AT3G09922 | ACTGCAGAAGGCTGATTCAGACT | AAGCTTGCCAAAGGATAGAAGTTG |
| AT5G03545 | GAGCGATGAAGATTGCATGAAG | GATCGAAGTTGCCCAAACGA |
| AT5G20150 | GCTGCCTTGCGGGTTTT | TTTAACTGTAGAGGCGGCAATG |
| XLOC_010238 | TTCGCGTCCTTTACGACATG | GCGAAAGAGTCCACCACCAT |
| XLOC_028295 | GTCCCCTGTTTCGAGGCATT | TCTTACCGTGGCTTTCAACAAG |
| XLOC_014746 | CCGTATGGATTCCAAAATACTCAA | AAGCCGCGACACGCTTT |
| XLOC_003815 | TGAGCTTGGCTGGTTTTGC | GAAGCCACCGAAGTTCTATCCA |

## Table S4. Correlation between P– regulated miRNAs and potential target mimics

| **miRNA** | **Target** | **PCC ^a^** | **Target score ^b^** | **Cluster ID ^c^** |
| --- | --- | --- | --- | --- |
| miR399a | XLOC_016349 | -0.18 | 3 | 6 |
| miR399b | XLOC_013840 | -0.63 | 4.5 | 6 |
| miR399c-3p | XLOC_013840 | -0.53 | 4.5 | 6 |
| miR399e | XLOC_016349 | -0.35 | 3 | 6 |
| miR156j | XLOC_029476 | -0.51 | 4 | 3 |
| miR169a-3p | XLOC_015007 | -0.31 | 2.8 | 6 |
| miR169d | XLOC_016349 | -0.22 | 4.2 | 6 |
| miR169e | XLOC_016349 | -0.26 | 4.2 | 6 |
| miR169f-5p | XLOC_016349 | -0.21 | 4.2 | 6 |
| miR169g-5p | XLOC_016349 | -0.26 | 4.2 | 6 |

**Note:**

a, PCC refers the expression correlation of miR399 and its target genes.

b, Target score is the target match score calculated by psRobot, and lower target score represents better base pair match between miRNA and targets. Then we filtered target mimics of Pi deficiency regulated miRNAs, as the method showed by Wu H et al (Wu *et al.* 2013). The predicted target mimics needed to meet the below criteria: i. perfect nucleotide pairing was required at the 5' end second to eighth positions of miRNA sequence; ii. bulges were only permitted at the 5' end ninth to 12th positions of miRNA sequence; iii. the bulge in eTMs should be composed of only three nucleotides; iv. except for the central bulge, the total mismatches within eTM and miRNA pairing regions should be no more than three.

c, The last column indicates the cluster that target genes or lncRNAs belonging to.

# References

**Gagliardi, D., Perrin, R., Marechal-Drouard, L., Grienenberger, J.M. and Leaver, C.J.** (2001) Plant mitochondrial polyadenylated mRNAs are degraded by a 3'- to 5'-exoribonuclease activity, which proceeds unimpeded by stable secondary structures. *The Journal of biological chemistry*, **276**, 43541-43547.

**Schuster, G., Lisitsky, I. and Klaff, P.** (1999) Polyadenylation and degradation of mRNA in the chloroplast. *Plant physiology*, **120**, 937-944.

**Wu, H.J., Wang, Z.M., Wang, M. and Wang, X.J.** (2013) Widespread long noncoding RNAs as endogenous target mimics for microRNAs in plants. *Plant physiology*, **161**, 1875-1884.
